# Astrocytes sense glymphatic-level shear stress through the interaction of sphingosine-1-phosphate with Piezo1

**DOI:** 10.1016/j.isci.2024.110069

**Published:** 2024-05-21

**Authors:** Antonio Cibelli, David Ballesteros-Gomez, Sean McCutcheon, Greta L. Yang, Ashley Bispo, Michael Krawchuk, Giselle Piedra, David C. Spray

**Affiliations:** 1 Department of Neuroscience, Albert Einstein College of Medicine, Bronx, NY 10461, USA

**Keywords:** Biological sciences, Neuroscience, Cell biology, Biophysics

## Abstract

Astrocyte endfeet enwrap brain vasculature, forming a boundary for perivascular glymphatic flow of fluid and solutes along and across the astrocyte endfeet into the brain parenchyma. We evaluated astrocyte sensitivity to shear stress generated by such flow, finding a set point for downstream calcium signaling that is below about 0.1 dyn/cm^2^. This set point is modulated by albumin levels encountered in cerebrospinal fluid (CSF) under normal conditions and following a blood-brain barrier breach or immune response. The astrocyte mechanosome responsible for the detection of shear stress includes sphingosine-1-phosphate (S1P)-mediated sensitization of the mechanosensor Piezo1. Fluid flow through perivascular channels delimited by vessel wall and astrocyte endfeet thus generates sufficient shear stress to activate astrocytes, thereby potentially controlling vasomotion and parenchymal perfusion. Moreover, S1P receptor signaling establishes a set point for Piezo1 activation that is finely tuned to coincide with CSF albumin levels and to the low shear forces resulting from glymphatic flow.

## Highlights

• Astrocytes respond to glymphatic-level shear stress when albumin is present
• Mechanism involves sphingosine-1-phosphate (S1P) sensitizing Piezo1 response to flow
• Ca^2+^ influx triggers downstream signaling, modulating parenchymal perfusion

## Graphical abstract

**Figure:**
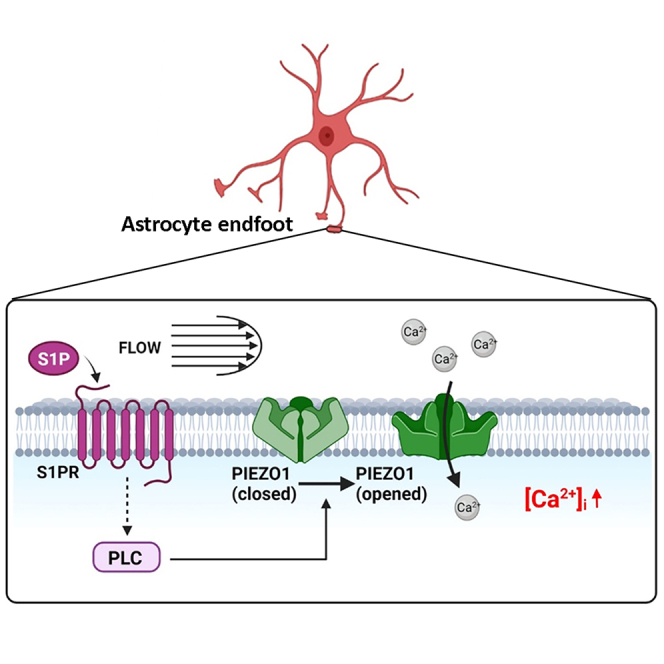

## Introduction

Neural cells are separated from the brain circulation by a multilayered barrier of vascular endothelial cells and smooth muscle or pericytes, a perivascular space that appears as thin basement membrane in electron micrographs, and astrocyte endfoot processes that enwrap the vessels.^1^ Astrocyte endfeet release vasoactive factors that control vessel tone and thus dictate local blood flow, a process termed functional hyperemia.^2^^,^^3^ They are enriched in channels and transport mechanisms that take up water and solutes and thereby drive perfusion of the brain parenchyma.^4^^,^^5^

Cerebrospinal fluid (CSF) flows from subarachnoid sinuses through perivascular spaces between vascular cells and astrocytes, forming the so-called glymphatic circulation.^6^^,^^7^ The topology, volume, and content of perivascular space through which the glymphatic circulation flows have been subjects of considerable experimental research and hydraulic modeling studies.^8^ Whereas electron microscopy has revealed a basement membrane between endothelial cells and astrocyte endfeet that is generally quite thin (<0.5 μm) and filled with dense matrix material,^9^ this narrow space reflects collapse as a consequence of fixation and its occlusion is at odds with functional studies showing dilated spaces for diffusion and rapid trajectories of perivascular nanoparticles.^8^ Forces on the astrocyte endfeet that are generated by the pulsations of vasculature and resultant flow within the perivascular space include strain imposed by vascular constriction and dilation, and interstitial flow between the endfeet from basement membrane to parenchyma.^10^ In addition, because the astrocyte endfoot creates a boundary for glymphatic flow, shear stress is expected to result from the flow trajectories that have been visualized by perivascular nanoparticle movements.^11^

Previous studies have measured astrocyte Ca^2+^ responses to vascular pressure gradients focally applied in acute brain slices^12^^,^^13^^,^^14^ and to brief concussive forces applied to astrocyte cell cultures,^15^^,^^16^ concluding that astrocytes are sensitive to mechanical stimuli but require relatively large forces in order to respond. However, astrocyte sensitivity to low-magnitude shear stresses corresponding to normal glymphatic flow has not been quantitatively examined. Moreover, these previous flow studies on astrocytes have used “artificial CSF” and other simple saline solutions that lack proteins such as albumin that are normally present at low concentrations in normal CSF. We have examined the sensitivity of astrocytes to low levels of mechanical stimuli using primary cultures of mouse astrocytes, in which we quantified intracellular Ca^2+^ responses to a range of calibrated shear forces in flow chambers with solutions containing levels of protein found in normal and pathological CSF. These experiments revealed that astrocytes are quite sensitive to shear stress when evaluated in the presence of physiological albumin levels. Notably, the threshold albumin concentration is very nearly the same as normal CNS protein levels, and the threshold force detected by astrocytes under these conditions is consistent with flow rates estimated from perivascular movements of nanoparticles *in vivo*. We conclude that astrocyte endfeet directly sense and respond to shear stress generated by glymphatic-level flow and that sensitivity to these low flow rates is greatly enhanced under conditions of heightened albumin levels that occur following breach of the blood-brain barrier, in response to infection, or consequent to autoimmune disorders.

## Results

### Properties of astrocyte cell cultures

Our goal was to quantify the shear stress sensitivity of astrocytes. In order to validate cell types in which Ca^2+^ changes responses were recorded, we immunostained primary cell cultures for the astrocyte intermediate filament protein glial fibrillary acidic protein (GFAP) and for the glial transcription factor Sox9. As illustrated in Figure S1A, most cells exhibited prominent GFAP staining when evaluated at passages 1 and 3 (82.14% ± 1.7%; 89.3% ± 1.2%); data pooled in histogram from 34 fields of view in Figure S1B. Similarly, the use of the Sox9 antibody indicated that the positive cells comprised 97.3% ± 0.5% of the population, suggesting a highly pure astrocyte culture (Figures S1C and S1D).

Additional evidence for preponderance of astrocytes in the cull cultures used for these experiments was obtained from studies in which we measured flow responses of astrocytes isolated from GFAPCre::GCaMP6f mice. As illustrated in Video S1 and in the selected frames shown in Figures S1E–S1G, virtually all cells showed increased CGaMP6f fluorescence in response to a range of shear flow rates (average of 5 regions of interest [ROIs] depicted in Figure S1G shown in Figures S1H and S1I). Display of Ca^2+^ changes at higher temporal resolution in five selected ROIs (Figures S1H and S1I) showed that cells responded to moderate and low flow rates with transient Ca^2+^ increases. Activity patterns in cells within clusters were similar to those of nearby but non-contacting cells (ROIs for selected traces are shown in Figures S1H and S1I). These measurements indicate that virtually all cultured cells express the GFAP-driven genetically encoded Ca^2+^ indicator GCaMP6, confirming that the cells with similar morphology that we selected in Fura2 experiments were also astrocytic. Moreover, most cells responded to shear stresses over a wide range of intensities.

**Figure 1:**
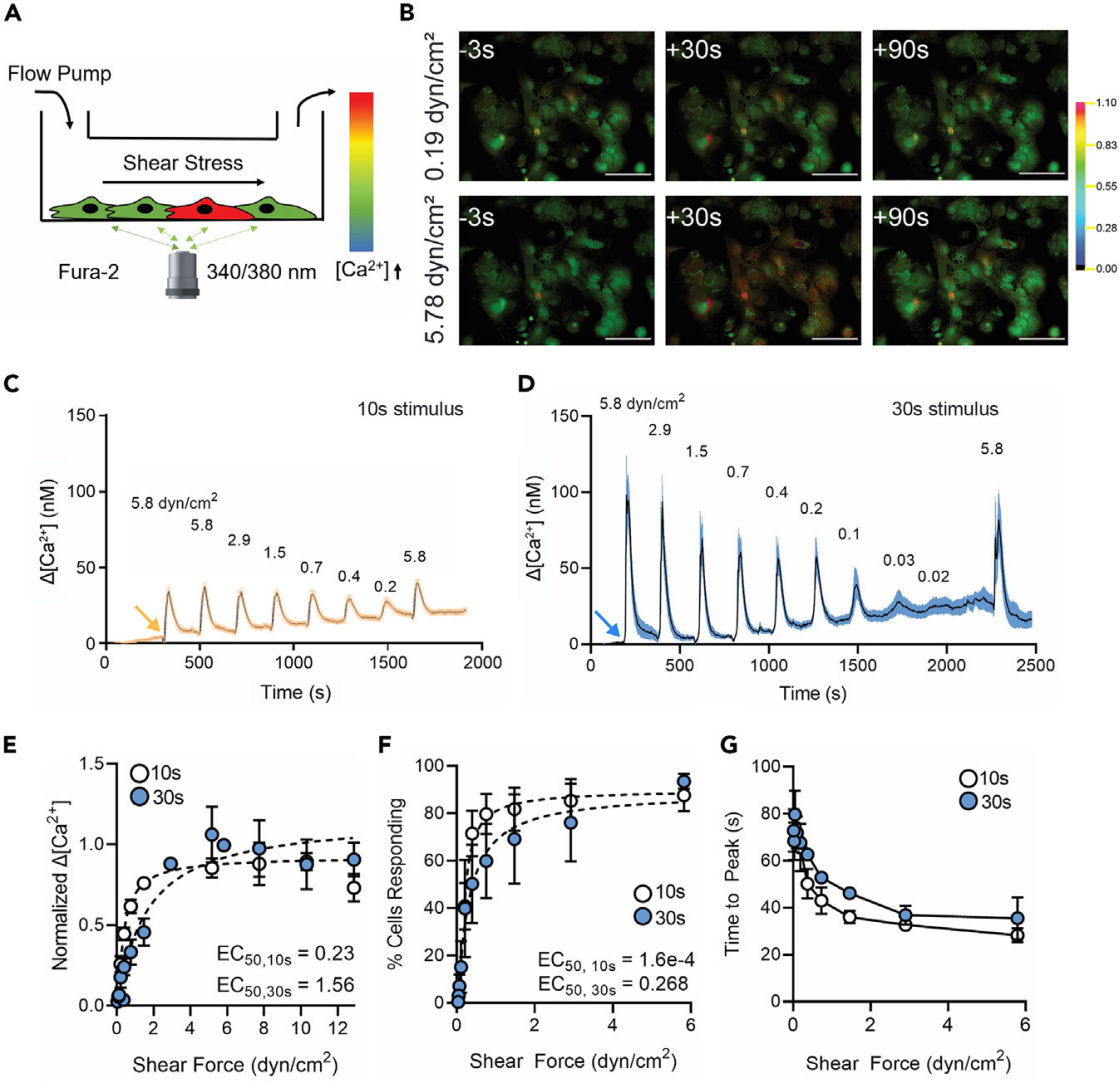
Astrocytes are highly sensitive to shear stress (A) Diagram of dual-wavelength Fura-2 imaging of astrocytes in parallel flow chambers. (B) Representative ratiometric fluorescence micrographs of Ca^2+^ response time course in astrocytes at moderate (0.19 dyn/cm^2^) and high (5.78 dyn/cm^2^) shear forces. Scale bars, 100 μm. (C) Representative change in [Ca^2+^]_i_ for a series of shear stresses using 10 s stimuli and (D) 30 s stimuli. Orange and blue arrows indicate when the first shear force was applied. (E) Normalized change in Ca^2+^ concentration amplitude across shear rates with 10 and 30 s stimuli. (F) Percentage of cells responding to a range of shear rates for 10 and 30 s stimuli. (G) Time from stimulus onset to peak Ca^2+^ concentration across a range of shear stresses for 10 and 30 s stimuli. All data shown as mean ± SEM. *n* ≥ 3 experiments for all treatments.

Video S1. Flow responses of astrocytes isolated from GFAPCre:GCaMP6f mice, related to Figure 1 and S1

### Astrocytes are sensitive to low levels of shear stress

Although GCaMP6f is a bright and highly responsive Ca^2+^ indicator, its fluorescence is monitored at a single wavelength and thus is concentration dependent and modifiable by cell volume change during swelling or shrinkage. In order to quantify the intracellular Ca^2+^ elevations in astrocytes without this potential confound, we used the ratiometric Ca^2+^ indicator Fura-2, where emission is measured when exciting at two wavelengths straddling the isosbestic point. Wild-type primary cortical astrocytes in culture were exposed to calibrated shear stresses in the range of 0–20 dyn/cm^2^ for 10 or 30 s at intervals of 3 min using flow media containing 45 μM BSA. As in the case of GCaMP6 imaging, ratiometric image sequences revealed responses in most cells evoked by shear stress (images taken before, during, and after 30 s shear stresses of 0.19 and 5.78 dyn/cm^2^ are shown in Figure 1B). As illustrated by the display of responses averaged within single experiments assessed with either 10 (Figure 1C) or 30 s (Figure 1D) stimuli, responses to the longer stimulation were larger. For both stimulus durations, magnitude was dependent on stimulus intensity, with responses decreasing at stresses below about 3 dyn/cm^2^ but persisting at even very low flow rates. When normalized Ca^2+^ changes were plotted as a function of shear stress, attenuation was marked below about 1 dyn/cm^2^, with a somewhat lower EC50 for the briefer stimulus duration (Figure 1E). Percentage of total cells responding also revealed strong amplification of sensitivity over the range of shear stress from 0 to 1 dyn/cm^2^ for both 10 and 30 s stimuli (Figure 1F). An additional property of the response that was stimulus dependent was the time to peak; for both 10 and 30 s stimuli, time to peak was rather stable at about 40 s for stimulus intensities above 2 dyn/cm^2^ but was considerably longer for briefer stimuli (Figure 1G). It is notable that the time to peak greatly exceeded the 10 s stimulus duration, implying the existence of an intracellular process interposed between stimulus and response in addition to opening of a mechanosensitive channel.

We also examined the responses to stimuli of constant intensity but varied duration. As illustrated in Figures S2A–S2C, response amplitude more than doubled when duration was extended from 5 to 30 s, whereas the fraction of responding cells was only slightly and not significantly higher at 30 s, the longest duration tested, than at 2 s, the shortest duration.

### Albumin is required for astrocyte high mechanosensitivity to flow

Protein concentration in blood is 6–8 g/dL, of which more than half is serum albumin (>35 g/L or about 0.5–0.8 mM), and its concentration is generally about 20 times lower in CSF.^17^^,^^18^ The usual reference interval for CSF total protein is 15–45 mg/dL (less frequently given as mg%), although protein concentrations are considerably higher in neonates and healthy elderly adults (as high as 400 mg/dL). Albumin is typically at least 60% of total CSF protein, and in the classic reference paper,^19^ concentration in normal adult brain was reported to be about 22 mg/dL. In this study, we have specified albumin concentration using molar equivalents, where 10–40 mg/dL corresponds to 1.5–6 μM (note that high normal levels might reach almost 60 μM).

**Figure 2:**
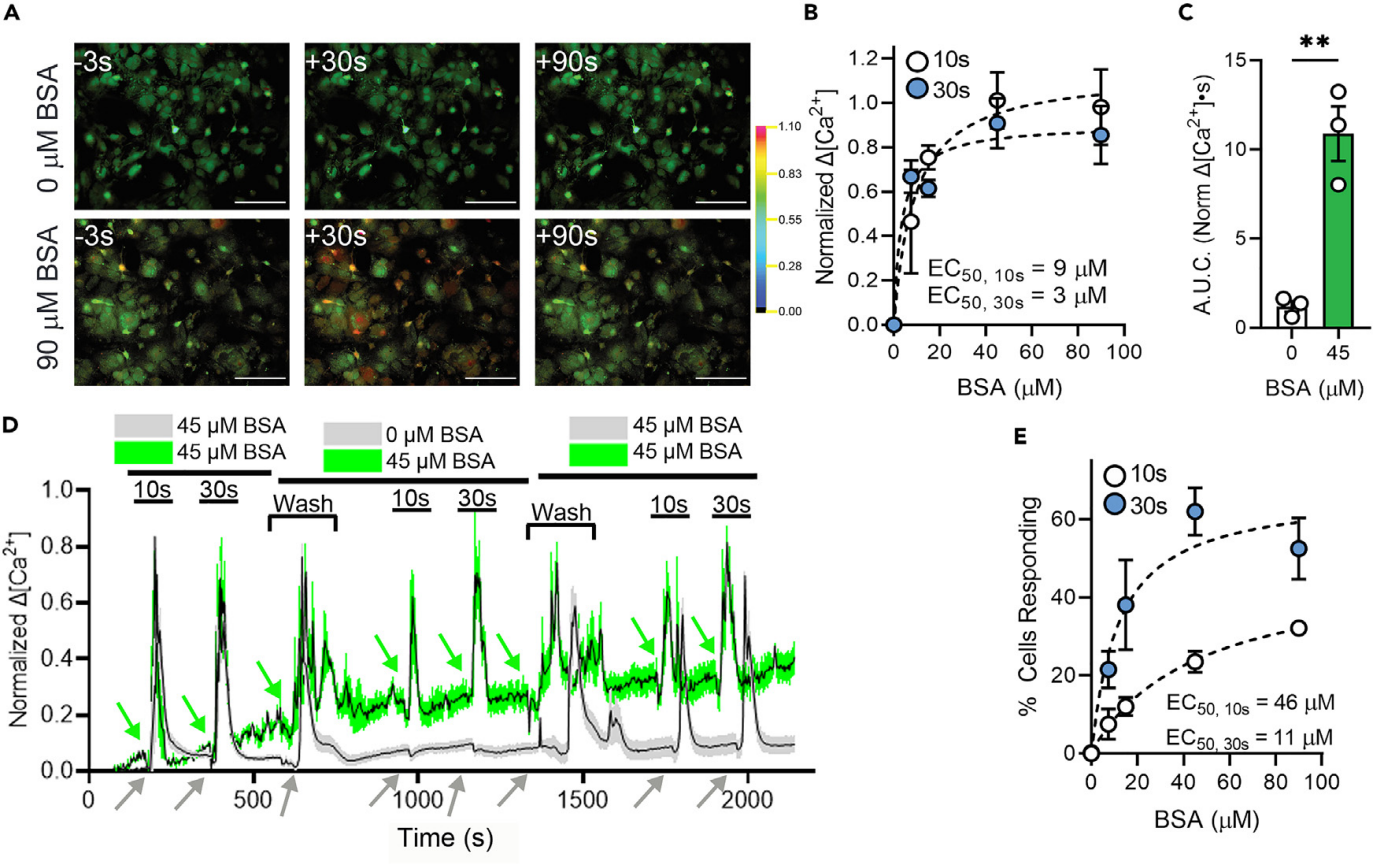
Astrocyte response to shear stress is dependent on albumin concentration (A) Representative ratiometric fluorescence micrographs of Ca^2+^ response time course in astrocytes at 0 and 90 μM BSA using 2 mL/min flow rate (2.57 dyn/cm^2^). Scale bars, 100 μm. (B) Normalized change in Ca^2+^ concentration amplitude across shear rates with 10 and 30 s stimuli. (C) Area under normalized Ca^2+^ response curve (AUC) for 30 s stimuli in 0 and 45 μM BSA. Unpaired Mann–Whitney test: ∗∗*p* < 0.01. (D) Representative traces of normalized change in Ca^2+^ concentration in 0 and 45 μM BSA. Green trace indicates experiment in which cells were alternately exposed to 10 and 30 s stimuli in the presence of 45 μM BSA, then valve was switched to another syringe containing the same BSA concentration, stimuli repeated, valve switched back to original syringe and stimuli repeated. Note that responses to each stimulus (denoted by arrows) are similar when BSA concentration is the same. Gray trace shows experiment in which 0 and 30 s stimuli were repeatedly applied, first in 45 μM BSA, then after switching to 0 μM BSA and then switching back. Note that responses to stimuli (denoted by arrows) in media lacking BSA are severely attenuated and that this is reversed by return to 45 μM. (E) Percentage of cells responding to a range of shear rates for 10 and 30 s stimuli. All data shown as mean ± SEM. *n* ≥ 3 for all experiments.

We examined the impact of albumin on astrocyte mechanosensitivity, testing a range of concentrations encompassing those to which astrocyte endfeet are exposed under physiological or pathophysiological conditions. For these experiments, cells were stimulated repeatedly with 10 and 30 s duration 2.8 dyn/cm^2^ forces, first in normal flow solution (45 μM albumin), then in test albumin concentrations (0–100 μM), followed by stimulation after rinse with normal flow solution (representative images for responses to 0 and 90 μM albumin shown in Figure 2A; measured fluorescence intensities in ROIs in single experiments for 10 and 30 s duration stimuli delivered in flow media containing 0 or 45 μM albumin shown in Figure 2D). When normal flow solution (containing 45 μM albumin) was exchanged for solution containing 15 μM or higher albumin concentration, astrocyte Ca^2+^ responses to either 10 or 30 s duration stimuli did not change in amplitude (Figure 2B) or in fraction of responding cells (Figure 2E), indicating that 15 μM or slightly lower is the concentration at which the response is maximal. Responses to lower concentrations of albumin were smaller in amplitude (Figures 2B–2D), and in the absence of albumin or other protein, the response to shear stress stimulation was almost completely abrogated (shown in each panel of Figure 2; note that when area under the curve for responses at 30 s was computed, the difference in responses was more than 10-fold). EC50 values calculated for amplitudes of responses to constant shear intensity and duration in different albumin concentrations ranged from 3 to 10 μM (Figure 2B) and from about 10 to 50 μM when fraction of responding cells was quantified (Figure 2E). Thus, astrocyte sensitivity to flow is vastly amplified by albumin, with EC50 in the range from 0.3 to 1 μM. As discussed, it is remarkable that this set point is within the range of protein concentrations that are present in normal CSF.^17^

### S1P receptors mediate amplification of astrocyte calcium signaling in response to shear stress

Shear stress responses of endothelial, smooth muscle, and other cell types is highly dependent on the presence of serum albumin, an effect that has been attributed to direct maintenance of the cell’s glycocalyx and more recently to the presence of sphingosine-1-phosphate (S1P) carried by the serum protein.^20^^,^^21^ To test whether albumin sensitization of astrocyte responsiveness is also dependent on S1P, we evaluated whether response in the absence of serum was restored by addition of S1P to BSA-free flow medium and whether sensitivity was reduced in the presence of S1P receptor inhibition.

**Figure 3:**
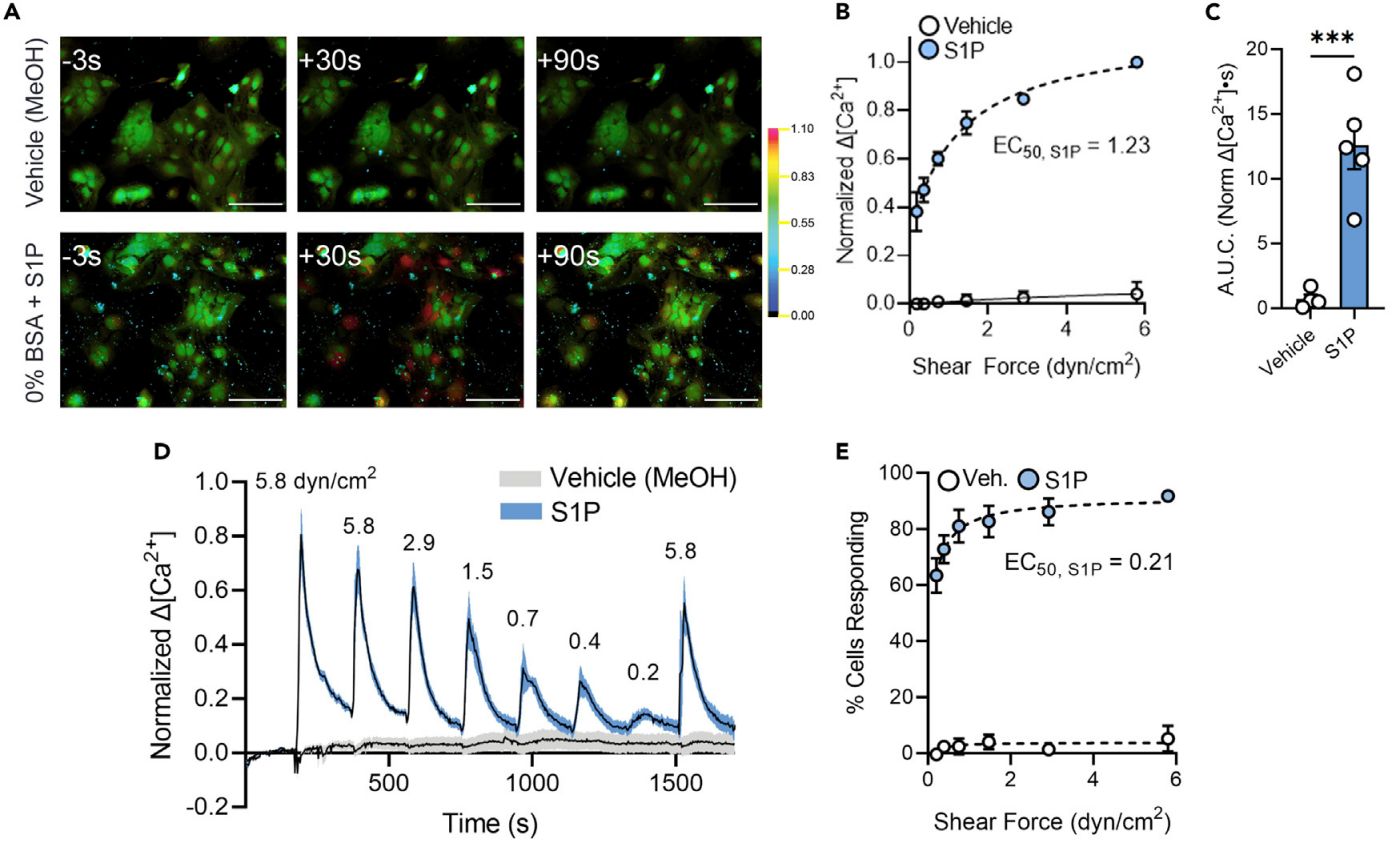
S1P recovers astrocyte mechanosensitivity in albumin-free condition (A) Representative ratiometric fluorescence micrographs of Ca^2+^ response time course in astrocytes in 0 μM BSA plus vehicle (methanol) and 0 μM BSA plus S1P across a range of shear stresses. Scale bars, 100 μm. (B) Normalized change in Ca^2+^ concentration amplitude across shear rates with 10 s stimuli. (C) Area under normalized Ca^2+^ response curve for vehicle control and after 1 h incubation prior to and for the duration of flow experiments with S1P conditions at 5.8 dyn/cm^2^. Unpaired Mann–Whitney test: ∗∗∗*p* < 0.001. (D) Representative traces of normalized change in Ca^2+^ concentration in 0 μM BSA with vehicle or S1P. (E) Percentage of cells responding to a range of shear rates for 10 s stimuli. All data shown as mean ± SEM. *n* ≥ 3 for all experiments.

To test directly for an effect of S1P on astrocyte mechanosensitivity, we treated cells with 1 μM S1P for at least 1 h in the absence of albumin, a condition under which astrocytes are normally unresponsive (see Figure 2). Cells were exposed to flow media lacking BSA, to which they did not respond, and then S1P was added and shear stress was reapplied. As shown in image frames in Figure 3A taken before, during and after stimulation at 5.8 dyn/cm^2^, responses were absent in flow medium lacking BSA but were restored in the presence of S1P. Amplitudes of responses and number of responding cells were both analyzed to quantify the impact of S1P on stimulus-response relations, which demonstrated a clear restoration of mechanosensitivity relative to 0% BSA vehicle control. In the presence of S1P, shear rate dependence of Ca^2+^ peak amplitude (EC50 = 1.23 dyn/cm^2^, Figures 3B and 3D) and percentage of responding cells (EC50 = 0.21 dyn/cm^2^, Figure 3E) were consistent with responses obtained in the presence of BSA (compare Figure 2). Overall responsiveness, evaluated by calculation of area under the curve in response to maximal stimulus (5.8 dyn/cm^2^, 10 s), was similar to that obtained in the presence of 45 μM BSA (compare Figure 3C with Figure 2C).

**Figure 4:**
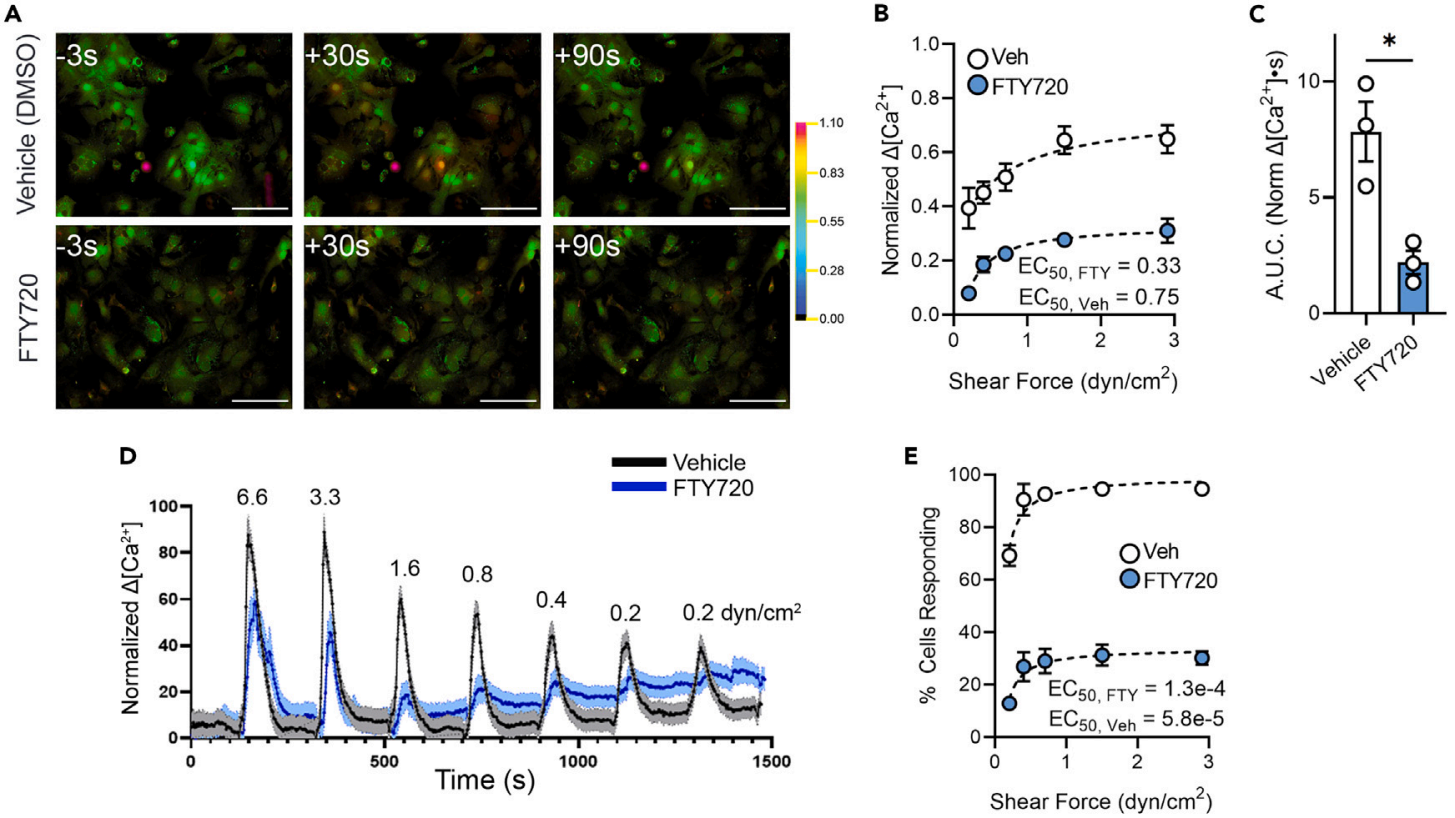
S1P receptor antagonist, FTY720, blunts astrocyte response to shear stress (A) Representative ratiometric fluorescence micrographs of Ca^2+^ response time course in astrocytes in 45 μM BSA plus vehicle (DMSO) and 45 μM BSA plus FTY720 in response to 10 s 2.9 dyn/cm^2^ shear. Scale bars, 100 μm. (B) Normalized change in Ca^2+^ concentration amplitude across shear rates with 10 s stimuli. (C) Area under normalized Ca^2+^ response curve for vehicle control and FTY720 conditions at 2.9 dyn/cm^2^. Unpaired Mann–Whitney test: ∗*p* < 0.05. (D) Representative trace of normalized change in Ca^2+^ concentration in 45 μM BSA with vehicle or FTY720. (E) Percentage of cells responding to a range of shear rates for 10 s stimuli. All data shown as mean ± SEM. *n* ≥ 3 for all experiments.

To evaluate the effect of inhibiting S1P receptor activity, we applied the broad-spectrum S1P receptor modulator fingolimod (FTY720). After incubation with 1 μM of FTY720 for 1 h, astrocyte responses to flow were substantially blunted (Figure 4A). The decreased response amplitude was evident across the entire range of stimulus intensities (Figures 4B and 4D), with only slightly reduced EC50 values compared to vehicle control. Area under the curve in response to 2.9 dyn/cm^2^ was also significantly reduced by FTY720 (Figure 4C, as was the percentage of responding cells at all stimulus intensities (Figure 4E)).

### Piezo1 is the target of S1P-amplified astrocyte shear stress sensitivity

To identify the mechanosensitive channel underlying astrocyte shear stress sensitivity, we tested effects of pharmacological inhibitors on astrocyte responses. These compounds, their presumed molecular targets, and tested concentrations are listed in Table 1. As shown in Figures S4 and S5, several inhibitors reduced response amplitude by >20%, including the nonspecific mechanoreceptor blocker gadolinium and the Ca-ATPase inhibitor cyclopiazonic acid, one of several tested TRPV4 antagonists, suggesting that they may participate in astrocyte shear stress responses. TRPV4 involvement in shear stress sensitivity of astrocytes was tested using three antagonists (RN-1734, HC064074, GSK2193874). As shown in Figure S4, acute exposure to 10–30 μM RN-1734 partially and reversibly reduced shear-induced responses, whereas the other antagonists were ineffective. To further test for a role of TRPV4 channels in shear stress sensitivity of astrocytes, we added the TRPV4 agonist 4α-PDD in the absence of albumin; astrocytes were unresponsive until albumin was restored (Figures S4G and S4H), indicating that TRPV4 activation alone was insufficient to activate the mechanosensitivity.

**Table 1:** List of the chemical compounds studied

Reagent	Source	Cat #	Concentration	Target
Bovine serum albumin (BSA)	VWR	10791-790	7.5, 15, 45, 90 μM	CSF protein
Sphingosine-1-phosphate	Sigma	S9666	1 μM	S1P receptor
FTY 720	Sigma	SML0700	1 μM	S1PR antagonist
Chondroitinase	Sigma	C2905	0.2 U/mL (8.33 mM)	ECM enzyme
Cyclopiazonic acid (CPA)	Sigma	C-1530	10 μM	ER calcium-ATPase inhibitor
Gadolinium (III)	Sigma	439770	1 mM, 30 μM	Calcium channel blocker
Yoda 1	Tocris	5586	10, 20 μM	PIEZO1 activator
U-73122	MCE	HY-13419	5 μM	PLC inhibitor
GsMTx4	Tocris	4912	1 μM	PIEZO1 blocker
Heparinase III	Sigma	H8891	0.25 U/mL (120 μM)	ECM enzyme
RN 1734	Tocris	3746	10, 20, 30 μM	TRPV4 antagonist
HC064074	Tocris	4100	1, 10 μM	TRPV4 antagonist
GSK2193874	Tocris	5106	1, 2 μM	TRPV4 antagonist
4α-Phorbol 12,13- didecanoate (4α-PDD)	Sigma	P8014	1 μM	TRPV4 agonist
Dooku1	Cayman Chemical Company	39161	10nM	Yoda 1 antagonist

**Figure 5:**
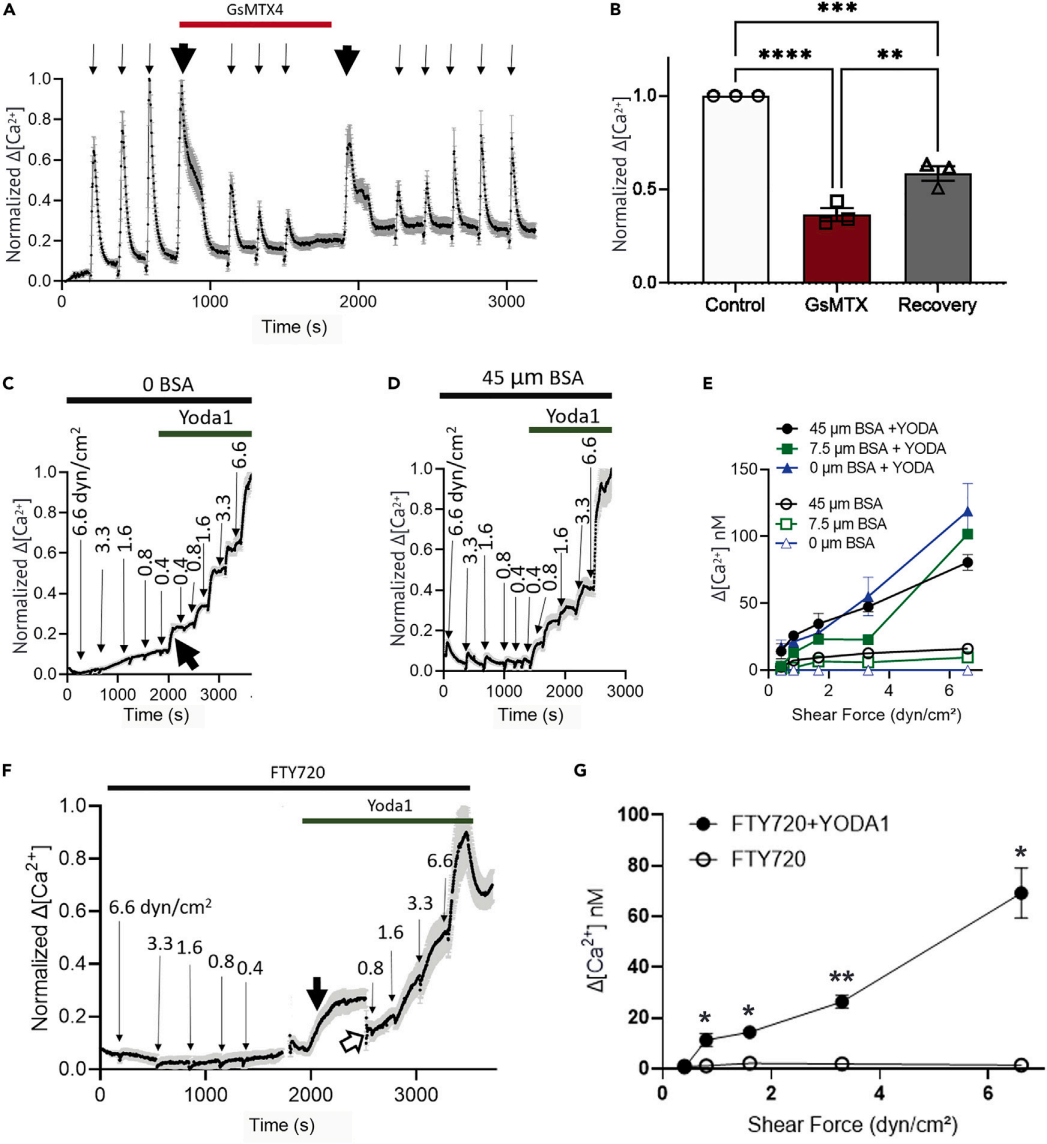
PIEZO1 determines the mechanosensitivity of astrocytes (A) Representative experiment showing that GsMTx4 (1 μM), a PIEZO1 antagonist, reversibly inhibits the astrocyte calcium responses to flow. 5.8 dyn/cm^2^ shear flow for 10 s (thin arrows) was applied at 300-s intervals before, after drug additions and after washout. Wash-in and washout of drug (thick arrows) were done at 0.2 mL/min for 2 min. (B) Summary of 3 independent experiments showing the reduction of shear-induced response by 1 μM GsMTX4 (red bar) and partial recovery (gray bar) upon washout. One-way ANOVA followed by Tukey’s post hoc test: ∗∗*p* < 0.01; ∗∗∗*p* < 0.001; ∗∗∗∗*p* < 0.0001. (C and D) Representative experiments showing the amplification of astrocyte calcium shear response by Yoda1, a PIEZO1 activator. A descending series of 10-s shear forces was applied in flow medium containing 0 or 45 μM BSA (C and D, respectively), then 10 μM Yoda1 was added and an ascending series of 10-s shear forces was applied. Shear forces (dyn/cm^2^) are indicated above each response. Note: substantially enhanced responses in the presence of Yoda1. (E) Summary data comparing Yoda1 effect in various BSA concentrations (0, 7.5, and 45 μM) at moderate shear forces. Note: significant enhancement of sensitivity in the presence of Yoda1 at all BSA concentrations. Two-way mixed ANOVA: 45 μM BSA with vs. without Yoda: *p* value = 0.0024; F(1,13) = 14.15; 7.5 μM BSA with vs. without Yoda: *p* value = 0.0021; F(1,21) = 12.28; 0 μM BSA with vs. without Yoda: *p* value = 0.0202; F(4,28) = 3.468. (F) Representative experiment of calcium response of astrocytes incubated in FTY720 for 1.5 h. A descending series of 10-s shear forces was applied in flow medium containing 45 μM BSA, then 10 μM Yoda1 was added (bold black arrow) and an ascending series of shear forces was applied; open arrowhead indicates refocusing. (G) Summary of 3 independent experiments showing the block of flow-induced stress after FTY720 incubation and the recovery of response upon the addition of Yoda1. Unpaired Student’s t test: ∗*p* < 0.05; ∗∗*p* < 0.01. *n* ≥ 3 for all experiments. All data shown as mean ± SEM.

Among the most potent of the inhibitors tested was the tarantula toxin, GsMTx4, which produced a blockade that was progressive as subsequent stimuli were applied following its wash-in and was gradually restored upon washout (representative experiment in Figure 5A, summary of results in Figure 5B). To further test the involvement of Piezo1 in the astrocyte shear stress responses, we applied Yoda1, an agonist that enhances Piezo1 activity. In these experiments, we first conducted a series of shear stress stimulations under conditions of 0, 7.5, and 45 μM BSA, and then washed in Yoda1 and repeated the series of shear stresses. As shown in typical experiments for 0 and 45 μM BSA in Figures 5C and 5D, and quantified from all experiments in Figure 5E, addition of Yoda1 strongly potentiated the response to shear stress, even enabling responses at all flow levels that were normally absent in solution lacking BSA (Figures 5C and 5E). To test the hypothesis that Yoda1 was acting to enhance activation of Piezo1 through S1P receptors, we repeated experiments in the presence of FTY720. As illustrated in Figure 5F and quantified in Figure 5G, shear stress responses were absent in the presence of FTY720 but responses to even very small shear stresses were rescued by treatment with Yoda1. The amplification of shear stress sensitivity by Yoda1 provides strong evidence that Piezo1 contributes substantially to the observed mechanosensitivity. Enhancement of responses by Yoda1 in the presence of the S1P receptor antagonist indicates that Piezo1 activation is downstream of S1P receptor signaling.

The potentiation of shear stress sensitivity by treatment with Yoda1 was long-lasting at even low stimulus intensities, leading to sustained rise in Ca^2+^ when multiple stimulus intensities were evaluated. In order to determine if Yoda1 led to elevation of intracellular Ca^2+^ in the absence of shear stress, we measured responses in static cultures under control conditions of 45 μM BSA and 10 μM Yoda1. As shown in Figure S6A for representative experiments and summarized in Figure S6B for all experiments at 1,000 and 2,000 s after Yoda1 treatment, there were no significant differences between treatment groups, indicating that mechanical stimulation is required for the response.

In order to test further for a role of Piezo1 in the amplification of the shear stress sensitivity, we tested whether the exaggerated responses in the presence of Yoda1 were affected by treatment with its specific small-molecule antagonist Dooku. As shown in Figures S7A and S7B, treatment with Yoda1 resulted in highly amplified responses to even very low shear forces; washing with Dooku restored the baseline and diminished but did not block response to flow.

### Inhibition of phospholipase C, a downstream target of S1PR activation, eliminates astrocyte shear stress-induced Ca^2+^ signaling

**Figure 6:**
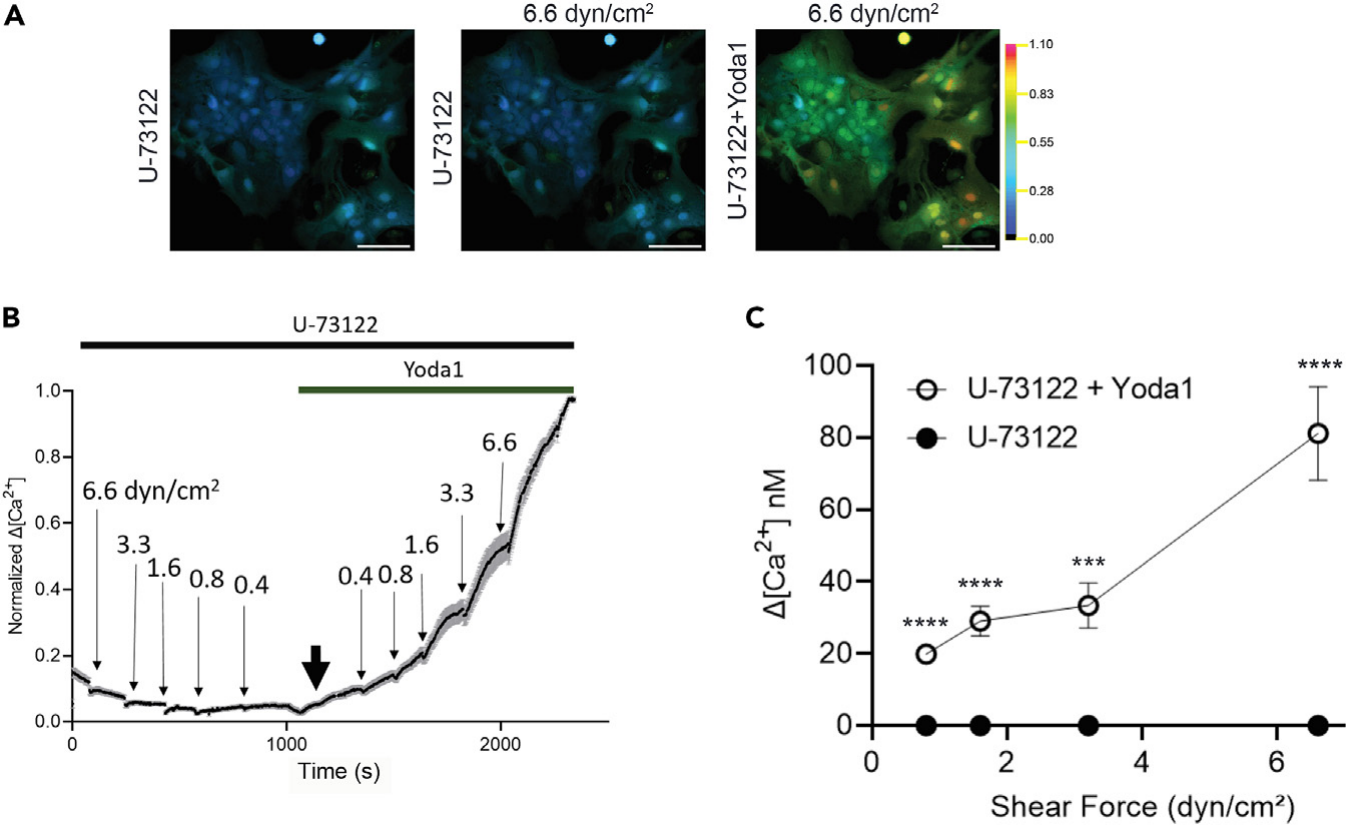
PLC inhibition eliminates astrocyte Ca^2+^ response (A) Representative ratiometric fluorescence micrographs of Ca^2+^ response time course in astrocytes in 45 μM BSA plus PLC inhibitor, U-73122, across a range of shear rates. Scale bars, 100 μm. (B) Representative traces of normalized change in Ca^2+^ concentration showing the blockade of shear response after incubation in U-73122 for 1.5 h. A descending series of 10-s shear forces was applied in flow medium containing 45 μM BSA and U-73122, then 10 μM Yoda1 was added (bold black arrow) and an ascending series of shear forces was applied. (C) Summary of 6 independent experiments showing the block of flow-induced stress after U-73122 incubation and the recovery of response upon the addition of Yoda1. Unpaired Student’s t test: ∗∗∗*p* < 0.001; ∗∗∗∗*p* < 0.0001. All data shown as mean ± SEM.

Blockade of response by fingolimod (FTY720) implicates G protein-coupled signaling through S1P receptors. FTY720 blocks S1PR 1, 3, 4, and 5, which signal through G proteins to activate phospholipase C (PLC), Ras, ROCK, and PI3 kinase. With the exception of the PLC inhibitor U-73122, drugs tested produced either slight or inconsistent inhibitions of astrocyte responses to flow media containing 45 μM albumin. After incubation in the PLC inhibitor for 1.5 h, however, astrocytes failed to respond to the full range of stimulus intensities (Figure 6C). In order to determine whether the effect of U-73122 was through the action on Piezo1 channels, we re-evaluated the responses following chemical activation of Piezo1 by Yoda1. As shown in Figures 6A–6C, responsiveness was absent in the presence of the PLC inhibitor but was rescued by treatment with Yoda1.

### Extracellular proteoglycan matrix participates in astrocyte mechanosensitivity

**Figure 7:**
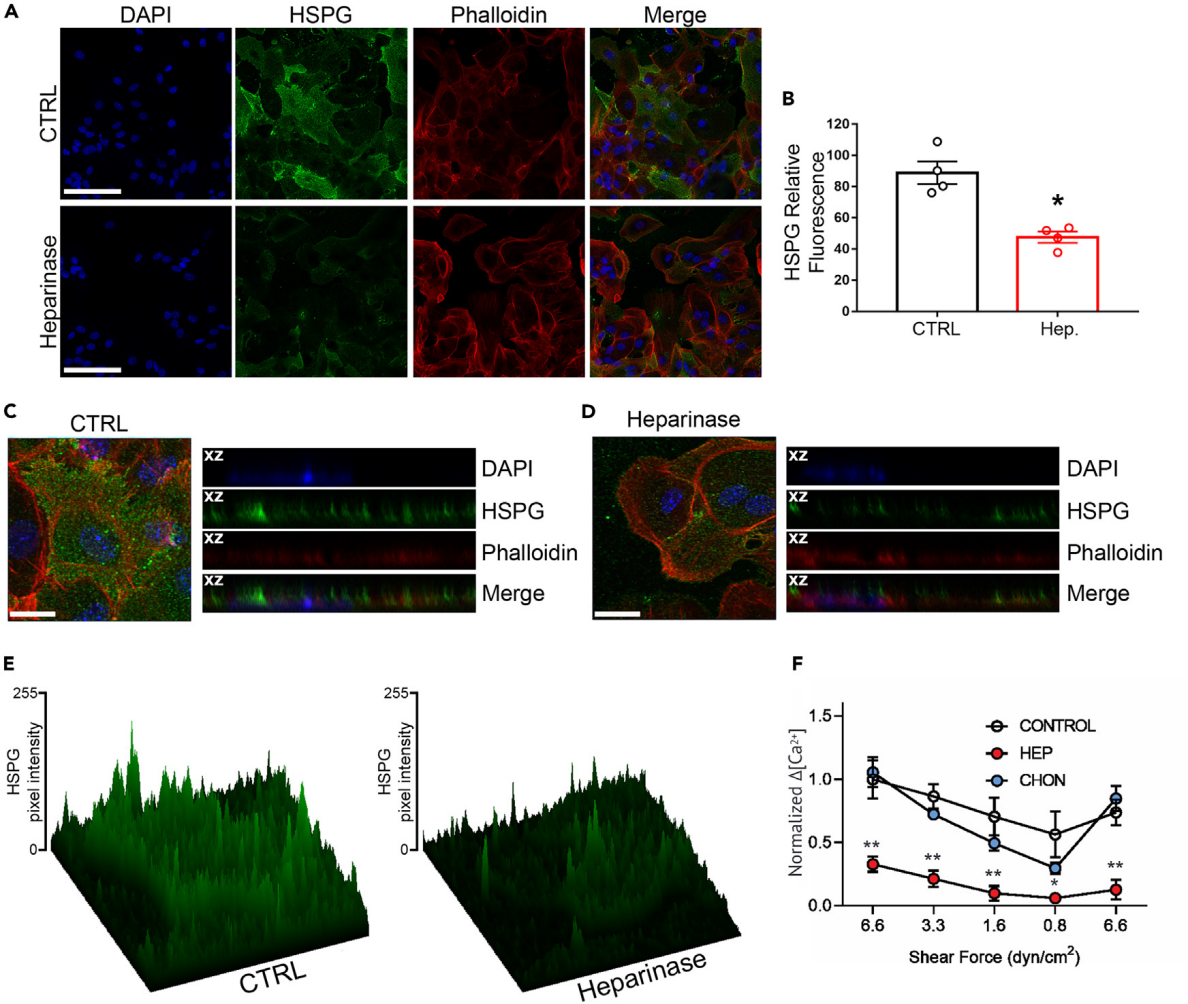
Attenuation of responses by heparan sulfate proteoglycan degradation (A) Representative single-slice confocal images of HSPG (green), F-actin cytoskeleton visualized with phalloidin (red) and their merge in WT astrocytes. Cells were cultured in DMEM without (CTRL) and with heparinase III (Heparinase) for 2 h. The nuclei were stained blue with DAPI. Scale bar, 50 μm. (B) Histogram showing HSPG fluorescence intensity quantification expressed as a percentage relative to that of the control (CTRL, black), considered as 100% after 2 h treatment with heparanase III (Hep., red). Data are expressed as mean ± SEM. Four images per experiment, from a total of four experiments, were taken for each condition. Unpaired Mann–Whitney test: ∗*p* < 0.05. (C) Left, confocal maximum intensity projections of control WT astrocytes immunostained for HSPG (green), F-actin (red), and nuclei (blue). Scale bar, 20 μm. Right, xz cross-sectional views of the stacked confocal images from the right showing the degree of cell surface distribution between HSPG (green), actin cytoskeleton (red), and nuclei (blue). (D) Left, confocal maximum intensity projections of WT astrocytes after 2 h treatment with heparanase immunostained for HSPG (green), F-actin (red), and nuclei (blue). Scale bar, 20 μm. Right, xz cross-sectional views of the stacked confocal images from the right showing the degree of cell surface distribution between HSPG (green), actin cytoskeleton (red), and nuclei (blue). (E) Reconstructed 3D surface plot images of (C) and (D). Color range from black to green indicates the relative level of fluorescence intensity in pixel. (F) Summary of normalized astrocyte calcium responses to a range of shear forces for control and after enzymes degrading heparan sulfate proteoglycan or chondroitin sulfate proteoglycan. *n* = 3, 4, 8 for control, HSPG degradation, CSPG degradation, respectively. Unpaired Student’s t test: ∗*p* < 0.05; ∗∗*p* < 0.01. All data shown as mean ± SEM.

Astrocytes secrete and are attached to proteoglycans and glycosaminoglycans that fill interstitial space in the brain and provide a reservoir for lipophilic molecules, including S1P.^22^^,^^23^ Previous studies on endothelial and smooth muscle cells showed that shear stress responses in those cell types are greatly attenuated by enzymatic removal of the glycocalyx.^24^ In order to determine whether these extracellular space molecules play a role in astrocyte shear responses, we immunostained astrocyte cultures for three major components of the brain extracellular space: chondroitin sulfate proteoglycans (CSPG), laminin (Figure S8A), and heparan sulfate proteoglycans (HSPG). We then treated the cells with enzymes to degrade the proteoglycans and tested the consequence for astrocyte mechanosensitivity in flow media containing 45 μM albumin. As shown in Figures 7A–7C, 7E, S8A, S8B, and S8D, we found abundant surface expression of these extracellular matrix components on the astrocytes. For HSPG, treatment for 2 h with heparinase III decreased the expression by 53% ± 3.5% (Figures 7B and 7E). Treatment with chondroitinase for 2 h produced a similar degree of loss of CSPG (Figures S6B–S6D). As shown in Figure 7F, treatment with heparanase dramatically attenuated response to all intensities of shear stress, whereas CSPG cleavage had minimal effect on the responsiveness.

## Discussion

### Forces that the endfoot feels

Astrocytes are exposed to diverse mechanical forces, including rhythmic pressure differentials in CSF driven by the cardiac cycle, traumatic forces in concussive brain injury or multiple mild impacts as in soccer heading, interactions with substrates of varying stiffness, as in glial scars, as well as membrane stretch due to osmotic volume changes and stretch due to vasomotion. Previous studies have measured astrocyte Ca^2+^ responses to focal pressure pulses,^25^ pressures across the arteriolar wall,^12^^,^^13^^,^^14^ and brief high-intensity concussive forces.^15^^,^^16^^,^^26^^,^^27^^,^^28^^,^^29^ In each of these studies, sensitivity was quite low.

However, astrocyte endfeet form a cylindrical perivascular boundary in which shear forces would be expected to be generated by flow of the perivascular glymphatic circulation^6^ along the vascular cells. Thus, even low flow through the perivascular space should generate shear stresses to which astrocytes may respond. Consistent with this hypothesis, we report here (Figure 1) that astrocytes respond to shear stresses well below 0.1 dyn/cm^2^ when measured using solutions approximating the composition of CSF. To put this finding into context, flow velocity measured from movements of injected nanoparticles in perivascular space^8^ permits calculation of shear stress in this compartment; from Figure 4C of that paper, dV/dt is about 12/s and shear stress at the wall is about 0.12 dyn/cm^2^ [Note that this calculation assumes that the perivascular space is empty; shear stress would be much higher if matrix were present and impeded flow.] We conclude that astrocytes are very responsive to mechanical force magnitudes to which their endfeet are normally exposed.

Other recent studies provide additional evidence that low-level mechanical stimuli in brain can have physiological effects. For example, treadmill running and passive head movements in rodents were reported to exert interstitial shear stress of 10–30 dyn/cm^2^ in prefrontal cortex,^30^ a force sufficient to internalize neuronal serotonin receptors. Low shear stress with protein-containing solution applied to cultured astrocytes (0.5 dyn/cm^2^) was shown to greatly enhance phagocytosis of lysed cells.^31^ CSF shunt implantation is common treatment for hydrocephalus, often failing due to development of astrocyte scar at regions of shear stress >0.5 dyn/cm^2.^^32^ Astrocytes in 2% FBS responded to 0.5 but not 0.05 dyn/cm^2^ shear stress with cytokine release.^33^ Moreover, gene expression in astrocytes is modified by both pressure-driven fluid shear stress and by electro-osmosis due to direct current as used in transcranial stimulation.^34^

### Enhancement of response by albumin

The shear stress sensitivity that we measured in astrocytes is much higher than that reported in most previous studies. One likely methodological difference is the use of simple saline solutions lacking protein in previous flow studies.^12^^,^^13^^,^^14^^,^^15^^,^^16^ Extensive studies of endothelial and osteocyte responses to shear stress^24^^,^^35^^,^^36^ have shown that shear response depends upon inclusion of albumin in flow medium, which is hypothesized to minimize glycocalyx shedding and maximize availability of lipid effectors.^23^ With regard to albumin concentration, it is important to note that although protein levels within CSF are vastly lower than in plasma, the levels found to amplify responses in our studies (from <1 to 9 μM, with EC50 values ranging from 0.3 to 1 μM) are within the critical normal range. Levels of CSF protein, consisting mostly of albumin, are about 8 μM in infants, about 15 μM in adults,^17^ and much higher following infarct or in conditions such as *neuromyelitis optica* and multiple sclerosis.^18^ Our studies indicate that shear stress sensitivity is modulated over this normal range of albumin concentrations, and maximal sensitivity occurs at levels seen following immune challenge.

Previous studies beginning more than 20 years ago revealed that astrocytes responded with Ca^2+^ elevation when exposed to albumin and that this response was abrogated by depletion of lipoproteins in the albumin.^37^^,^^38^ Recent studies on endothelial cells have shown that S1P preserved the shear stress responses that were otherwise abolished in the absence of albumin and that inhibition of S1P receptors by FTY720 (fingolimod) blunted mechanosensitivity in the presence of serum protein.^20^^,^^39^ We have found very similar effects on astrocyte responses to shear stress: S1P restored the blunted sensitivity in the absence of albumin (Figure 3) and treatment with the antagonist largely blunted shear responses in the presence of albumin (Figure 4).

To determine whether the amplification of the shear responses by S1P was mediated through S1P receptors, we applied the broad-spectrum antagonist FTY720 (fingolimod), which greatly attenuated response amplitude at all stimulus intensities. Blockade was maximally about 70% complete, indicating the possibility that additional S1P receptors that are less sensitive to this compound may also be involved, that partially cleaved matrix may remain functionally active, or that additional pathways are activated by flow in the presence of albumin. Nevertheless, these findings reveal that the potentiation of astrocyte mechanoresponsiveness by CSF albumin levels results in part from S1P, raising the questions of which downstream pathway is responsible and how albumin mediates S1P activation. Pharmacological experiments using the selective PLC inhibitor U-73122 achieved striking response blockade, implicating this pathway in the response, but the precise identification of downstream mediators requires additional studies using specific pharmacology and directed genetic manipulations.

### Role of extracellular matrix

Brain extracellular matrix contains relatively high amounts of glycosaminoglycans (including both chondroitin sulfate and heparin sulfate bound to protein) and relatively low amounts of collagens and fibronectin.^40^ We have reported that cultured astrocytes possess a glycocalyx consisting primarily of heparin sulfate proteoglycans linked to actin cytoskeleton^22^; in the same report, treatment with heparanase altered endothelial cell cytoskeleton. Our finding in this study that heparanase cleaved the glycocalyx and proportionately decreased mechanoresponsiveness is consistent both with the hypothesis that the glycocalyx plays a direct role in mechanotransduction and that it acts as a local reservoir for S1P and perhaps other lipid mediators.

### Role of Piezo1

Albumin in the perfusion solution, and presumably S1P in the albumin, greatly amplifies the astrocyte sensitivity to shear stress. This amplification is likely due to modulation of the shear stress-activated mechanosensitive channels. To identify which mechanosensor is required for shear transduction, we tested pharmacological inhibitors of known mechanoreceptors, namely the multimodal TRPV4 channel and the true mechanoreceptor Piezo1. Responses were slightly reduced by one of three TRPV4 receptor antagonists tested, suggesting possible contribution to the responses, although the ineffectiveness of the other antagonists implies the involvement of other processes. By contrast, the tarantula neurotoxin GsMTx4, a moderately selective Piezo1 inhibitor, rapidly and effectively blocked mechanical responses in astrocytes. Furthermore, the chemical Piezo1 activator Yoda1 greatly enhanced response sensitivity in both the presence and absence of albumin and in the presence of FTY720, conditions in which responses are normally absent. Repeated flow stimulation in the presence of Yoda led to summation of responses, possibly indicating flow-independent action on intracellular Ca^2+^. However, application of Yoda in an open chamber lacking shear stress did not evoke such increases in Ca^2+^ (Figures S6A and S6B), and application of the Yoda antagonist Dooku blocked Yoda’s enhancement of mechanical sensitivity without impairing normal shear responses (Figures S7A and S7B).

As drawn in the graphical abstract, we conclude from these studies that albumin in CSF contains S1P that binds to its receptor to activate PLC. PLC then amplifies the response of Piezo1 to shear stress, leading to elevated intracellular Ca^2+^ and other downstream responses. Response amplification by Yoda reveals that Piezo1 is downstream of and does not require S1P.

Constitutive sensitization of PIEZO1 by S1PR signaling sets the threshold for response to glymphatic circulation. It is remarkable that the set point for Piezo1 activation is shear force less than 0.1 dyn/cm^2^ in cells exposed to ≥8 μM albumin, values that are consistent with measured perivascular flow and normal CSF albumin concentration, as noted previously. Even very small variations in either force or CSF protein profoundly affected astrocyte responses in our studies. The rapidity by which protein removal blocked astrocyte responses indicates that the set point for PIEZO1 gating is constitutively maintained, and total inhibition strongly implicates the involvement of the downstream PLC pathway. S1PR signaling sets basal activity of ion channels in various cell types as has been shown previously, such as in stimulating vascular development through Src kinase stimulation of baseline Piezo1 activation^41^ and in mediating nociceptor sensitivity through regulation of KCNQ excitability by S1P.^42^ However, our study indicates the involvement of S1PR signaling in the astrocyte mechanosome and that its downstream pathway generates constitutive responsiveness of Piezo1.

### Limitations of the study

Quantitation of applied shear stress was critical for these studies, but it required that the studies be conducted on astrocytes cultured in calibrated flow chambers. Although we used primary astrocytes rather than immortalized cell lines, these studies used mouse cells, which may differ in responses from their human counterparts. Moreover, the environment of the cells in culture differed from that at the blood-brain barrier *in vivo*. One major difference is that the astrocyte endfoot process is the principal surface exposed to perivascular flow in the intact brain, and consequently the sensitivity to mechanical simulation *in vivo* might be expected to be even higher than measured in our studies. Finally, our studies designed to identify signaling pathways responsible for the S1P-mediated sensitization relied on pharmacological agents selected for their efficacy and target specificity, but off-target actions and potential involvement of additional signaling pathways could not be totally ruled out.

Despite these limitations, our studies reveal that the astrocyte mechanosome responsible for the detection of shear stress includes S1P-mediated sensitization of the mechanosensor Piezo1. Fluid flow through perivascular channels delimited by the vessel wall and the astrocyte endfeet thus generates sufficient shear stress to elicit Ca^2+^ responses in astrocytes, thereby potentially controlling vasomotion and parenchymal perfusion. Moreover, S1P receptor signaling establishes a set point for Piezo1 activation that is finely tuned to coincide with albumin level in CSF and to the low shear forces resulting from glymphatic flow.

## STAR★Methods

### Key resources table

**Table:** 

REAGENT or RESOURCE	SOURCE	IDENTIFIER
**Antibodies**		

Mouse monoclonal anti-GFAP	Sigma	SAB5201104; RRID:AB_2827276
Rabbit polyclonal anti-Sox9	Sigma	AB5535; RRID:AB_2239761
Mouse monoclonal anti-HSPG	US Biological	H1890; RRID:AB_10013601
Rabbit polyclonal anti-Laminin	Santa Cruz	sc20142; RRID:AB_2133767
Mouse monoclonal anti-CSPG	Sigma	C8035; RRID:AB_476879
Phalloidin Alexa Fluor 594–conjugated	Thermo Fischer	A12381; RRID:AB_2315633
Donkey anti-mouse Alexa Fluor 488–conjugated	Invitrogen	A21202; RRID:AB_141607
Donkey anti-mouse Alexa Fluor 594–conjugated	Invitrogen	A-21203; RRID:AB_2535789
Donkey anti-rabbit Alexa Fluor 488- conjugated	Invitrogen	A-21206; RRID:AB_2535792

**Bacterial and virus strains**		

No bacterial and virus strains used	N/A	N/A

**Biological samples**		

No biological samples used	N/A	N/A

**Chemicals, peptides, and recombinant proteins**		

Phosphate buffered saline	Corning	21-030-CV
Trypsin	Gibco	25200–056
Dulbecco’s Modified Eagle’s Medium (DMEM)	Corning	10-014-CV
Fetal bovine serum	Thermo Fisher	10437–028
Penicillin-streptomycin	Gibco	15070–063
Bovine Serum Albumin	Sigma	A3059
Bovine serum albumin (30% stock)	VWR	10791–790
ProLong	Invitrogen	P36965
DAPI	Thermo Fisher	62248
Fura-2 a.m.	Thermo Fisher	F1221
Sphingosine-1-phosphate	Sigma	S9666
FTY 720	Sigma	SML0700
Chondroitinase	Sigma	C2905
Cyclopiazonic acid (CPA)	Sigma	C-1530
Gadolinium (III)	Sigma	439770
Yoda 1	Tocris	5586
U-73122	MCE	HY-13419
GsMTx4	Tocris	4912
Heparinase III	Sigma	H8891
RN 1734	Tocris	3746
HC064074	Tocris	4100
GSK2193874	Tocris	5106
4α-Phorbol 12,13- didecanoate (4α-PDD)	Sigma	P8014
Dooku1	Cayman Chemical	39161
Dimethyl sulfoxide	Sigma	D2438

**Experimental models: Cell lines**		

Primary cultures of neonatal murine astrocytes	N/A	N/A

**Experimental models: Organisms/strains**		

C57BL/6J mouse strain	Jackson Labs	000664
Gfap-Cre (B6.Cg-Tg(Gfap-cre) 73.12 Mvs/J) mouse strain	Jackson Labs	012886
GCaMP6f ^(sB6J.Cg−Gt(ROSA)26Sortm95.1(CAG−GCaMP6f)Hze/MwarJ)^ mouse strain	Jackson Labs	028865

**Software and algorithms**		

Leica Application Suite X	Leica Microsystems	https://www.leica-microsystems.com
Fiji/ImageJ	Fiji	https://imagej.nih.gov/ij/
GraphPad Prism 9	GraphPad Software LLC	https://www.graphpad.com
Metafluor	Molecular Devices	https://www.moleculardevices.com/
BioRender	BioRender	https://biorender.com

**Other**		

Ibidi-coated μ-SlideVI^0.4^	ibidi	80606
Falcon® Cell Culture Flask	Corning	353109
Leica DM6 B Fluorescence LED Motorized Microscope	Leica Microsystems	https://www.leica-microsystems.com
Leica DMi8 SP8 microscope	Leica Microsystems	https://www.leica-microsystems.com
Nikon TE 2000 microscope	Nikon	https://www.microscope.healthcare.nikon.com
Sutter filter changer	Sutter	https://www.sutter.com/

### Resource availability

#### Lead contact

Further information and requests for resources and reagents should be directed to and will be fulfilled by the lead contact Antonio Cibelli (antonio.cibelli@uniba.it).

#### Materials availability

This study did not generate new unique reagents.

#### Data and code availability

• Data reported in this paper will be shared by the lead contact upon request.
• This paper does not report original code.
• Any additional information required to reanalyze the data reported in this paper is available from the lead contact upon request.

### Experimental model and study participant details

#### Animals

All procedures described below were reviewed and approved by the Einstein Institutional Animal Care and Use Committee (IACUC), protocols #20190302 and 00001463. The procedures follow ARRIVE guidelines and are consistent with the Guide for the Care and Use of Laboratory Animals, 8th Edition. Mouse colonies were maintained in AALAC-accredited Laboratory Animal Resource Facility with a 12-h light/dark cycle (lights on at 7:00 a.m., light off at 7:00 p.m.) and with access to food and water *ad libitum*.

#### Primary astrocyte isolation and cell culture

Experiments were performed entirely on primary cultures of neonatal murine astrocytes. Perinatal (postnatal days 1–3) C57BL/6J (Jackson Labs #000664) or GCaMP6 male and female mice (crossbreeds of homozygous Gfap-Cre (B6.Cg-Tg(Gfap-cre) 73.12 Mvs/J), Jackson Labs #012886) and GCaMP6f ^(sB6J.Cg−Gt(ROSA)26Sortm95.1(CAG−GCaMP6f)Hze/MwarJ)^, (Jackson Labs #028865 strains) were euthanized via decapitation and cortical tissue was isolated by excision and removal of meninges in ice-cold phosphate buffered saline (PBS) (Corning #21-030-CV). Isolated cortices were digested in 0.05% trypsin (Gibco #25200-056) for 10 min. Digested tissue was replated in tissue culture treated flasks (Falcon 353109) in astrocyte media: DMEM (Corning #10-014-CV) supplemented with 10% fetal bovine serum (FBS) (Thermo #10437-028) and 1% penicillin-streptomycin (PS) (Gibco #15070-063). At 14 days, flasks were shaken at 180 rpm for 12-24 h in an incubated shaker (Ecotron HT) to remove residual tissue and microglia.^43^^,^^44^ Astrocytes were cultured up to passage 3 for use in experiments.

### Method details

#### Antibodies

The following primary antibodies were used: mouse monoclonal anti-GFAP (1:500; Sigma cat # SAB5201104), rabbit polyclonal anti-Sox9 (1:500, Sigma cat # AB5535), mouse monoclonal anti-HSPG (1:200; US Biological cat #H1890), rabbit polyclonal anti-Laminin (1:50; Santa Cruz cat # sc20142) and mouse monoclonal anti-CSPG (1:200; Sigma cat #C8035). Phalloidin Alexa Fluor 594–conjugated (1:1000; Thermo Fischer cat # A12381) was used to stain F-Actin.

The secondary antibodies used for immunofluorescence analysis were donkey anti-mouse Alexa Fluor 488–conjugated (1:1000; Invitrogen cat # A21202), donkey anti-mouse Alexa Fluor 594–conjugated (1:1000; Invitrogen cat # A-21203) and donkey anti-rabbit Alexa Fluor 488- conjugated (1:1000; Invitrogen cat # A-21206).

#### Immunofluorescence

To assess the relative purity of primary astrocytes culture, cells were fixed with 4% paraformaldehyde for 15 min, washed 3 times in PBS, and permeabilized with 0.3% Triton X-100. After blocking using 2% BSA for 30 min, cells were incubated for 2 h with primary antibodies at room temperature and washed with PBS + BSA. Cells were finally incubated with secondary antibodies and mounted with ProLong (Invitrogen, cat# P36965), and DAPI for nuclear staining.

To evaluate the removal of cell surface proteoglycans by treatment with heparinase III (0.25 U/mL for 2 h) or chondroitinase ABC (0.2 U/mL for 2 h), both controls and enzyme exposed cells were immunostained. Cells were chilled on ice and washed 3 times with ice-cold PBS. Cells were then blocked with 1% BSA and incubated on ice with primary antibodies for 2 h, followed by ice-cold PBS BSA rinses and fixation with 4% paraformaldehyde.^22^ Cells were finally incubated with secondary antibodies and mounted as described above.

#### Microscopy and image analysis

Epifluorescence images were obtained with a Leica DM6 B Fluorescence LED Motorized Microscope using a 40× HC PL Apo oil CS2 objective.

Confocal images were obtained with an automated inverted Leica DMi8 SP8 microscope using a 63× NA = 1.4 Oil PL APO, WD = 0.14 mm objective. All confocal images were collected using 594 and 488 nm laser lines for excitation and a pinhole diameter of 1 Airy unit. Images were taken serially from top to bottom of each cell field with a raster size of 1024 × 1024 in the x–y planes and a z-step of 0.2–0.5 μm between optical slices (typically 15–25 optical sections per stack relative to different protein staining). Leica Application Suite X (Leica Microsystems CMS GmbH) and Fiji-ImageJ software were used to construct and process three-dimensional (3D) surface plot images and projections from z stack.

The purity of primary astrocyte cultures was quantitatively analyzed by determining the overlap between DAPI-stained nuclei and the astrocyte markers GFAP or Sox9. The analysis was conducted on 3 to 5 different ROI from each of 3 independent experiments. All the cells in each field were counted using Fiji-ImageJ software and analyzed using GraphPad Prism 9.

To quantify the extent of HSPG and CSPG removal by proteases, immunostained-positive cells were identified and at least four different square regions of interest (ROIs; fixed squared 10 μm^2^) per cell were selected. The average protein fluorescence intensity profile for each ROI was plotted and measured by using Fiji-ImageJ software and analyzed using GraphPad Prism 9 software for statistical analysis.

#### Parallel plate flow experiments

Primary astrocytes up to passage 3 were plated in Ibidi-coated parallel plate flow chambers (μ-SlideVI^0.4^, ibidi #80606) 24–48 h prior to experiments and maintained in astrocyte media (see above). One h prior to testing, cells were loaded with 10 μM Fura-2 a.m. (Thermo #F1221) in astrocyte culture media. Post-incubation with Fura-2 a.m., flow chambers were connected to syringe pumps (New Era model NE 300) via 20 or 60 mL syringes and chambers were rinsed for 1–3 min at 0.3 mL/min with flow media. Flow solutions were prepared by dilution of bovine serum albumin (30% BSA stock; VWR #10791-790) to 0.3% (45 μM) or other desired concentration at the time of the experiment in extracellular solution containing 120 mM NaCl, 4 mM KCl, 2 mM CaCl_2_, 2 mM MgCl_2_, 10 mM HEPES, 10 mM D-glucose, pH 7.4 (https://cshprotocols.cshlp.org/content/2016/8/pdb.rec093229.short).

Applied shear stresses (ranging from <0.1 to >20 dyn/cm^2^) were calculated according to formulae in Application Note 11: Shear Stress and Shear Rates for ibidi μ Slides (ibidi GmbH, Version 4.1, 29 March 2016) and validated in initial experiments by weighing triplicate flow samples encompassing the range of flow rates used. For experiments involving change of solutions, flow was switched via stopcock valve between two syringe pumps, connected to the microfluidic chamber by low dead volume tubing. Cells were imaged by Nikon microscope (TE 2000, 20x 0.65 NA objective) with MetaFluor software (Molecular Devices) using Hamamatsu camera and Sutter filter changer connected to the microscope via a fiber optic cable. Intracellular Ca^2+^ was measured at 2 Hz by imaging regions of interest (ROIs) corresponding to individual astrocytes (diagrammed in Figure 1A) with 380/340 nm dual excitation (Sutter filter changer) and Fura-2 filter cube. Changes in cytosolic Ca^2+^ levels in ROIs were imaged by epifluorescence at 1 Hz. Ratiometric fluorescence values obtained from ROIs were translated into intracellular Ca^2+^ concentrations ([Ca^2+]^_i_) according to an *in vitro* calibration curve using Metafluor software.^45^ In some experiments, Ca^2+^ changes were measured as change in fluorescence intensity using 488 nm excitation and FITC filter cube in response to flow in astrocytes cultured from GFAPCreGCaMP6f mice (see Figure S1).

In addition to a few experiments examining high flow rates and in which we compared responses to varied stimulus duration at constant flow (Figure S2), two protocols were routinely used to quantify sensitivity to flow. In both protocols, stimuli were generally separated by 5 min intervals. Protocol 1 consisted of 10s or 30s exposures to moderate flow (4.5–5 mL/min), followed by progressive 2-fold flow decreases to 0.1 mL/min or less, followed by another 4.5–5 mL/min stimulus (corresponding to a range from 5.8 to 6.6 to <0.2 dyn/cm^2^ as labeled in raw data figures and analysis) at 3 min intervals. As illustrated in Figure S3, similar responses were observed regardless of whether flow rates were decreased from high to low or increased from low to high. Protocol 2 consisted of paired moderate flow stimuli presented in a sequence of two 10 s or alternating 10s and 30s durations, wash in/out BSA or drug at 0.3 mL/min for 2 min, paired stimulation, wash in/out BSA or drug at 0.3 mL/min for 2 min, then paired stimulation (See Figure 2D). BSA experiments were performed with BSA concentrations of 0, 0.05, 0.1, 0.3, and 0.6% (0–90 μM) using both Protocols 1 and 2.

#### Drug experiments

To test the role of sphingosine-1-phosphate (S1P) in astrocyte sensitivity to flow, cells were treated for 1–2 h prior to and for the duration of flow experiments with 1 μM S1P receptor antagonist fingolimod (FTY720) (stock 1 mM in DMSO, Sigma #SML0700). FTY720 experiments were performed with Protocol 1 in flow media supplemented with 0.3% BSA. Conversely, the ability to reverse the nonresponsive phenotype for astrocytes in 0% BSA was tested by > 1 h incubation prior to and for the duration of flow experiments with 1 μM S1P (stock 0.2 mM in 1:5 methanol: water, Sigma #S9666) in 0% BSA flow media using Protocol 1, 10 s. Results were compared to those obtained with vehicle alone (DMSO or methanol).

To test whether the mechanosensitive channel Piezo1 was involved in the responses of astrocytes to flow, we tested effects of an inhibitor (the spider toxin GsMTX4; Tocris #4912 stock 50 μM in DMSO) and the Piezo1 channel activator Yoda1 (Tocris 6586 10 and 20 μM, stock 20 mM in DMSO).

Mediation of astrocyte shear stress sensitivity through the phospholipase C (PLC) pathway was tested through pharmacological inhibition. Cells were treated with 5 μM of the PLC inhibitor U-73122 (MedChemExpress Cat #: HY-13419) after incubation for 1.5 h prior to and for the duration of flow experiments and tested using Protocols 1.

The role of extracellular glycocalyx in amplification of astrocyte mechanotransduction was tested by degrading chondroitin sulfate proteoglycan (CSPG) with 0.2 U/mL chondroitinase ABC (Sigma #C2905-2UN) and heparan sulfate proteoglycan with 0.25 U/mL heparanase III (Sigma #H8891). Cells were treated for 1–2 h at 37°C prior to flow experiments in flow media containing 45 μM BSA and tested using Protocol 1, 10 s, with enzyme omitted from the flow media. Other drugs used are listed in Table 1.

### Quantification and statistical analysis

#### Data analysis

Fluorescence ratios were generally quantified in >20 individual ROIs in each experiment, and values presented in histograms represent means and standard errors from three or more independent experiments on astrocytes obtained from separate mice. Representative raw data traces displayed as figures are averaged from ≥5 selected ROIs within a single experiment. Parameters measured included amplitude of peak response (change in [Ca^2+^] from baseline value, actual value or normalized to maximal response), fraction of responding cells (# ROIs with Ca^2+^ change >5% baseline/total # ROIs), time to peak (from >5% baseline change to peak), and area under the curve (AUC: change in Ca^2+^ over time above baseline).

#### Statistics

Multiple ROIs were selected in each field for calculation of Ca^2+^ levels in flow experiments. Mean values obtained in individual experiments were averaged between multiple experiments on different cell cultures to obtain mean and variance values for comparison of amplitudes, number of responding cells and area under curve, as presented. Statistical significance was evaluated using unpaired Mann–Whitney test for non-normally distributed data, while unpaired Student’s t test, One-way ANOVA (Holm-Sidak’s and Tukey’s multiple comparisons test), and two-way ANOVA were performed for normally distributed data as described in figure legends using GraphPad Prism 9. Nonlinear Regression was used to calculate EC_50_ using GraphPad Prism 9. *p*-Values are defined as follows: ∗*p* < 0.05, ∗∗*p* < 0.01, ∗∗∗*p* < 0.001, ∗∗∗∗*p* < 0.0001. All data are presented as mean ± SEM.
