# Supplementary material for: Astrocytes sense glymphatic-level shear stress through the interaction of sphingosine-1-phosphate with Piezo1
Source: iScience. 2024 May 21;27(6):110069. doi: 10.1016/j.isci.2024.110069 (PMC11167526; doi:10.1016/j.isci.2024.110069)
Supplement: Document S1. Figures S1–S8 [file mmc1.pdf]

iScience, Volume *27*

Supplemental information

Astrocytes sense glymphatic-level shear stress through the interaction of sphingosine-1-phosphate with Piezo1

Antonio Cibelli, David Ballesteros-Gomez, Sean McCutcheon, Greta L. Yang, Ashley Bispo, Michael Krawchuk, Giselle Piedra, and David C. Spray

SUPPLEMENTAL INFORMATION

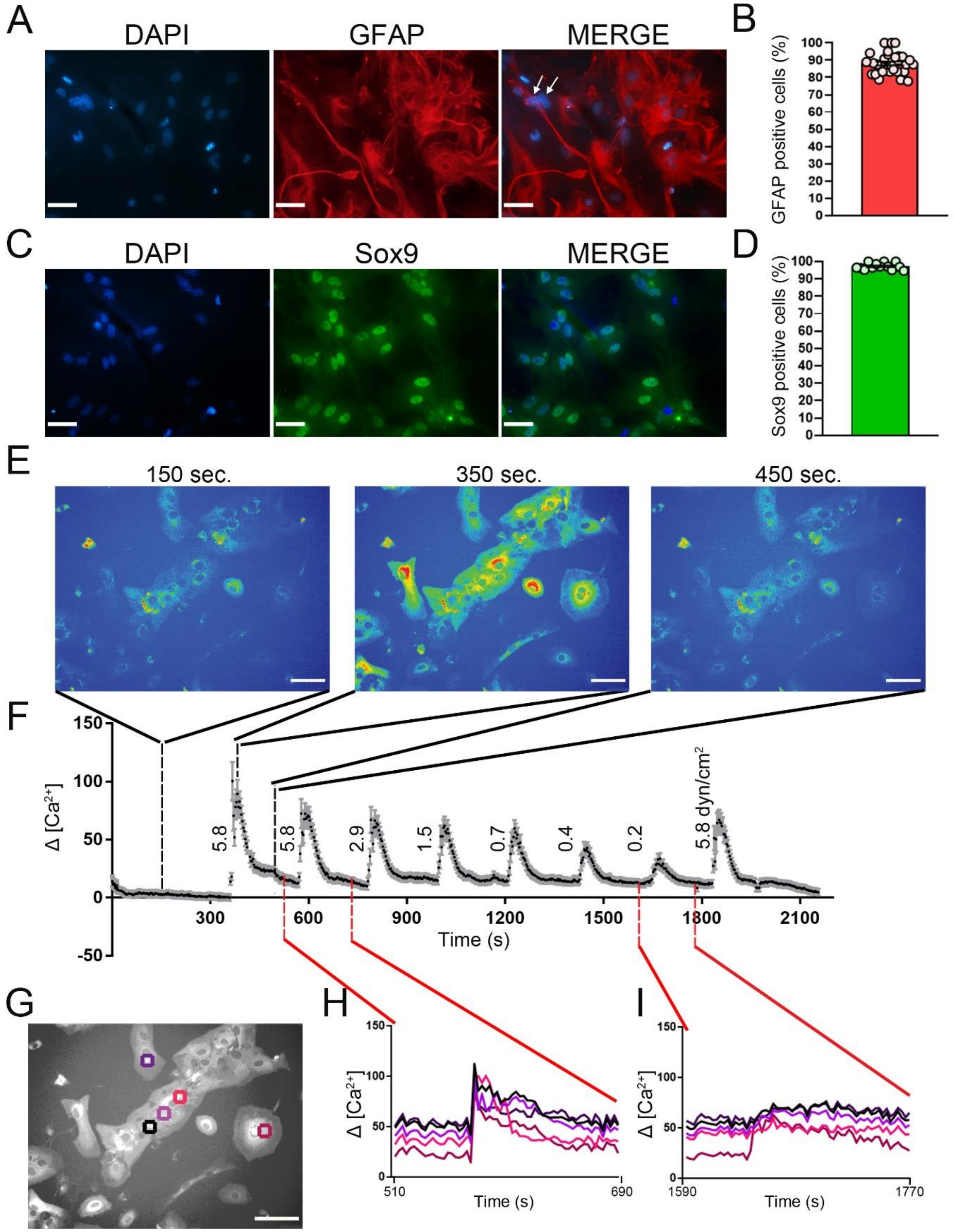
A
DAPI
GFAP
MERGE
B
GFAP positive cells (%)
C
DAPI
Sox9
MERGE
D
Sox9 positive cells (%)
E
150 sec.
350 sec.
450 sec.
F
Δ [Ca2+]
5.8
5.8
2.9
1.5
0.7
0.4
0.2
5.8 dyn/cm2
Time (s)
G
H
Δ [Ca2+]
Time (s)
I
Δ [Ca2+]
Time (s)

Supplemental Figure 1: *Primary astrocytes cultured in flow chambers are highly homogeneous as judged by GFAP and Sox9 expression in WT and Ca^{2+} responses in GFAPCre::GCaMP6f cells, related to Figure 1. (A) Epifluorescence micrographs showing GFAP immunostaining; GFAP shown in red and DAPI shown in blue. Arrows indicate GFAP negative cells. Scale bars 50 µm. (B) Percentage of cells in cultures stained positive for GFAP at passages 1 and 3 (data pooled from 34 regions of interest (ROIs) in 4 culture dishes; displayed as mean ± SEM. (C) Epifluorescence micrographs showing Sox9 immunostaining; Sox9 shown in green and DAPI shown in blue. Scale bars 50 µm. (D) Percentage of cells in cultures stained positive for Sox9 at passages 1 and 3 (data pooled from 25 regions of interest (ROIs) in 3 culture dishes; displayed as mean ± SEM. (E) Frames from a representative pseudo-colored GCaMP imaging experiment (where blue indicates lowest and red highest fluorescence levels, which are proportional to intracellular calcium levels in these GFAPCre::GCaMP6f astrocytes) showing average changes occurring before, during and after application of 30 sec 5.8 dyn/cm^2 stimuli. Scale bar 100 µm. Graph in (F) represents the averaged values obtained within the five ROIs throughout the entire field of view in response to a series of 30 sec exposures to shear force magnitudes indicated alongside the responses, ranging from 5.8 to 0.2 dyn/cm^2. (G) Regions of interest (ROIs) showing responses of adjacent and nonadjacent astrocytes to flow. Scale bar 100 µm. Traces of cell responses to high (H) and low (I) magnitude stimuli correspond to colors of ROIs. n=3.*

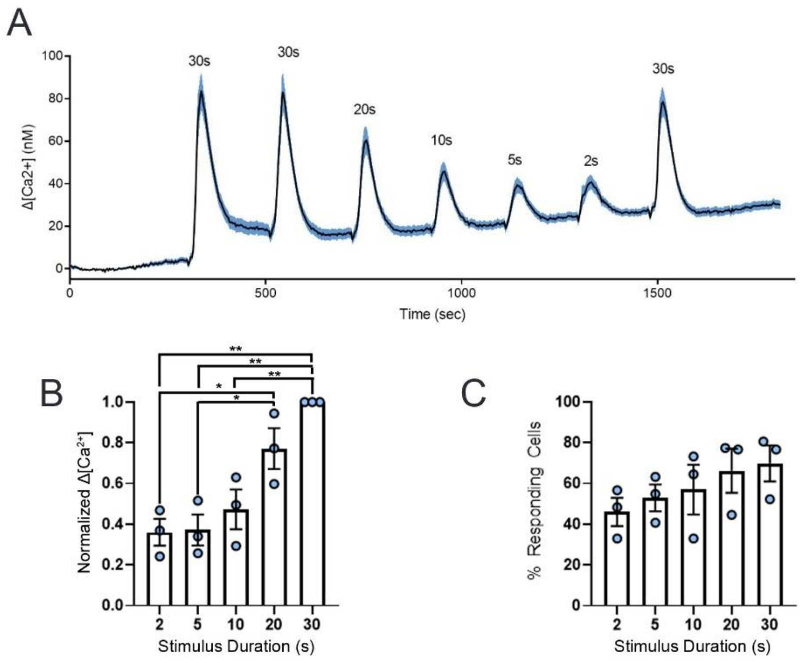

*Supplemental Figure 2: Astrocyte shear stress response is dependent on duration of stimulus, related to Figure 1 (A) Representative trace of Ca^{2+} responses to 5.8 dyn/cm^2 stimuli over a range of 2-20 sec stimulus durations. (B) Histogram showing the amplitude of calcium response as a function of stimulus duration. One-way ANOVA followed by Tukey's post hoc test: *p<0.05; **p<0.01. (C) Histogram showing the percentage of responding astrocytes as a function of stimulus duration. One-way ANOVA followed by Tukey's post hoc test. All data shown as mean ± SEM. n = 3 for all experiments.*

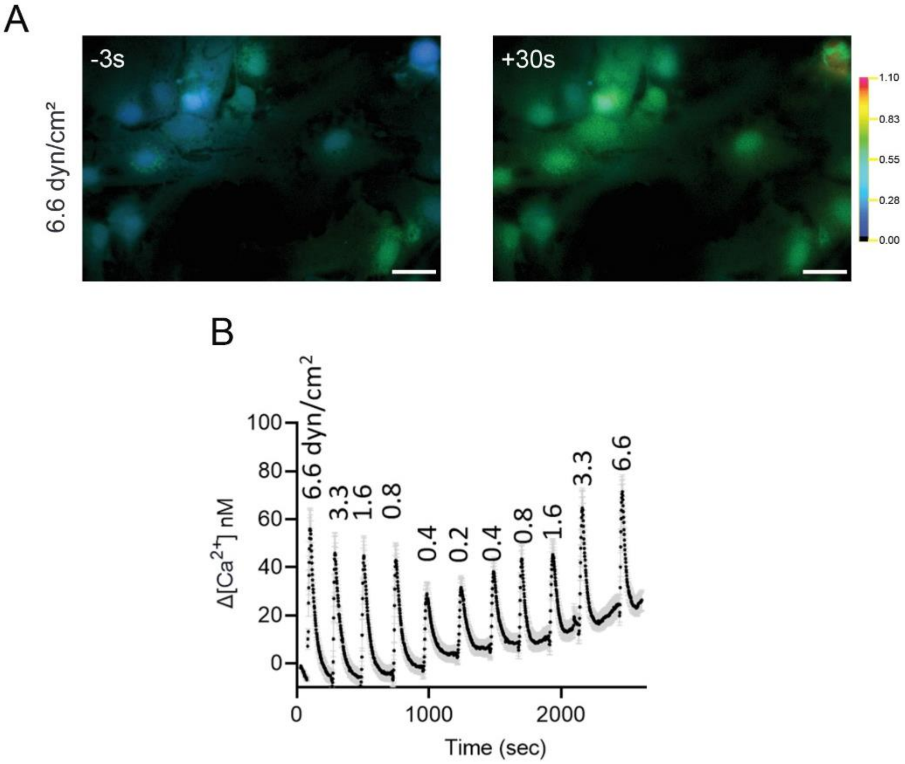

Supplemental Figure 3: Astrocyte shear stress response is independent of the order by which stimuli are applied, related to STAR Methods. (A) Representative ratiometric fluorescence micrographs of Ca^{2+} response time course in astrocytes at high (6.6 dyn/cm2) shear forces. Scale bar, 50 μm. (B) Representative traces of Ca^{2+} response over a range of stimulus intensities.

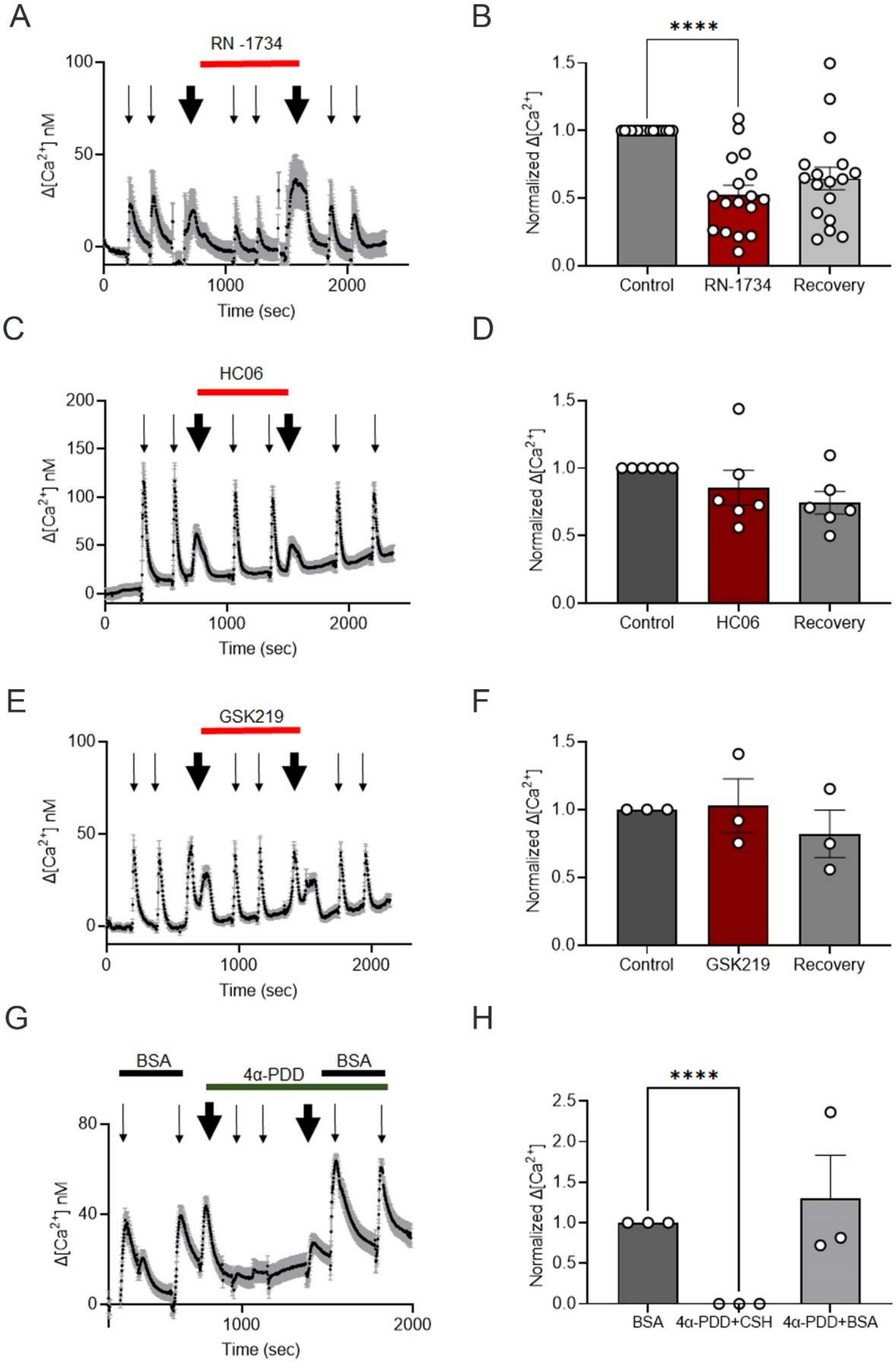
A
RN -1734
Δ[Ca2+] nM
100
50
0
0
1000
2000
Time (sec)
B
****
Normalized Δ[Ca2+]
1.5
1.0
0.5
0.0
Control
RN-1734
Recovery
C
HC06
Δ[Ca2+] nM
200
150
100
50
0
0
1000
2000
Time (sec)
D
Normalized Δ[Ca2+]
1.5
1.0
0.5
0.0
Control
HC06
Recovery
E
GSK219
Δ[Ca2+] nM
100
50
0
0
1000
2000
Time (sec)
F
Normalized Δ[Ca2+]
1.5
1.0
0.5
0.0
Control
GSK219
Recovery
G
BSA
4α-PDD
BSA
Δ[Ca2+] nM
80
40
0
1000
2000
Time (sec)
H
****
Normalized Δ[Ca2+]
2.5
2.0
1.5
1.0
0.5
0.0
BSA
4α-PDD+CSH
4α-PDD+BSA

*Supplemental Figure 4: Effects of TRPV4 agonist and antagonists on flow responses, related to Figure 5. (A) Representative traces of change in $[Ca^{2+}]i$ showing the shear responses after acute exposure to RN-1734, a TRPV4 inhibitor. Shear forces at 5.8 dyn/cm^2 for 10 seconds were applied at 300 sec intervals (thin arrows). RN-1734 was applied and removed at a flow of 0.2 ml/min for 3 minutes (thick arrows). (B) Summary of 17 independent experiments showing the partial reduction of shear-induced response by 10-30 µM RN-1734 (red bar) and the partial recovery (gray bar) upon washout. One-way ANOVA followed by Tukey's post hoc test: ****$p \leq 0.0001$. (C) Representative traces of change in $[Ca^{2+}]_i$ showing the lack of change in shear response after acute exposure to TRPV4 antagonist HC064074. Shear forces at 5.8 dyn/cm2 for 10 seconds were applied at 300 sec intervals (thin arrows). HC064074 was applied and removed at a flow of 0.2 ml/min for 3 minutes (thick arrows). (D) Summary of 6 independent experiments showing the lack of effect on shear-induced response by 10 µM HC064074 (red bar) and the recovery (gray bar) upon washout. (E) Representative traces of change in $[Ca^{2+}]i$ showing the lack of change in shear response after acute exposure to 2 µM GSK2193874, another TRPV4 inhibitor. Shear forces at 5.8 dyn/cm^2 for 10 sec were applied at 300 sec intervals (thin arrows). GSK219 was applied and removed at a flow of 0.2 ml/min for 3 minutes (thick arrows). (F) Summary of 3 independent experiments showing the shear-induced response by GSK219 (red bar) and the recovery (gray bar) upon washout. (G) Representative traces of change in $[Ca^{2+}]i$ showing that acute exposure to TRPV4 agonist 4α-PDD in the absence of BSA did not evoke response, which was recovered upon addition of BSA. Shear forces at 5.8 dyn/cm^2 for 10 sec were applied at 300 sec intervals (thin arrows). 4α-PDD and BSA were exchanged at a flow of 0.2 ml/min for 3 min (thick arrows). (H) Summary of 3 independent experiments showing the lack of response in the absence of BSA despite the presence of 4α-PDD, and the recovery of response in the presence of BSA and 4α-PDD (gray bar). One-way ANOVA followed by Tukey's post hoc test: ****$p \leq 0.0001$. All data shown as mean ± SEM.*

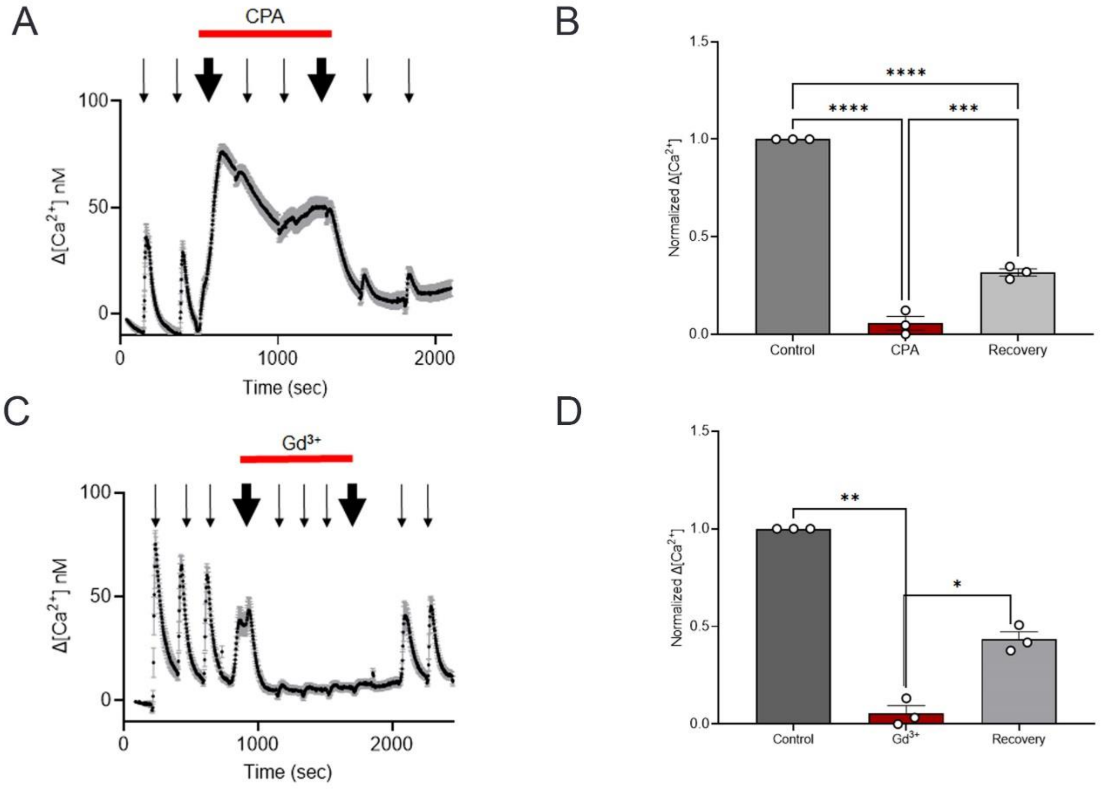

---

*Supplemental Figure 5: CPA and gadolinium potently inhibit calcium responses, related to Figure 5. (A) Representative traces of changes in $[Ca^{2+}]i$ showing the blockade of shear response after acute exposure to CPA. Shear forces at 5.8 dyn/cm^2 for 10 sec were applied at 300 sec intervals (thin arrows). CPA was applied and removed at a flow of 0.2 ml/min for 3 min (thick arrows). (B) Summary of 3 independent experiments showing significant reduction of shear-induced response by 10 µM CPA (red bar) and the partial recovery (gray bar) upon washout. One-way ANOVA followed by Tukey's post hoc test: ***p<0.001; ****p<0.0001. (C) Representative traces of changes in $[Ca^{2+}]i$ showing the complete inhibition of shear response after acute application of gadolinium. Shear forces at 5.8 dyn/cm2 for 10 sec were applied at 300 sec intervals (thin arrows). Gadolinium was applied and removed at a flow of 0.2 ml/min for 3 min (thick arrows). (D) Summary of 3 independent experiments showing significant reduction of shear-induced response by 1 mM gadolinium (red bar) and the partial recovery (gray bar) upon washout. One-way ANOVA followed by Tukey's post hoc test: *p<0.05; **p<0.01. All data shown as mean ± SEM.*

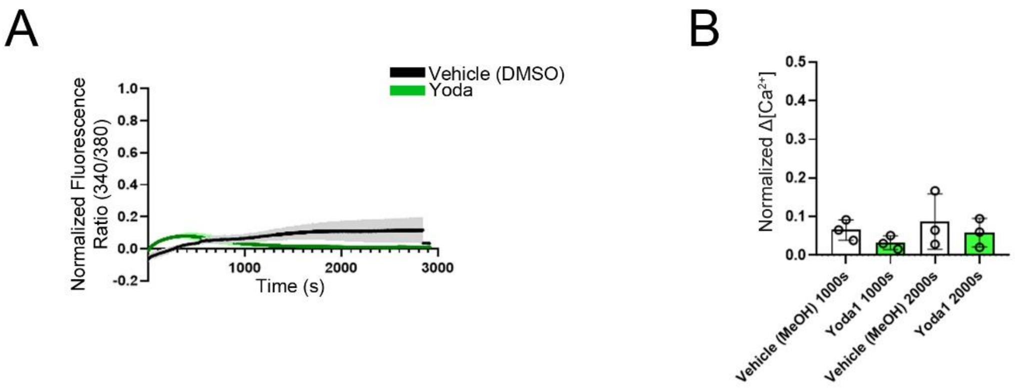

Supplemental Figure 6: Effect of Yoda1 in static cultures, related to Figure 5. (A) Averaged traces of normalized change in Ca^{2+} concentration in 45 μM BSA with vehicle or plus Yoda1 in the absence of shear stress. (B) Summary of 3 independent experiments showing the lack of effect on the elevation of intracellular Ca^{2+} by 10 μM Yoda1 (green bar) and 45 μM BSA with vehicle (white bar) in the absence of shear stress after 1000 and 2000 sec. One-way ANOVA followed by Tukey's post hoc test. All data shown as mean ± SEM.

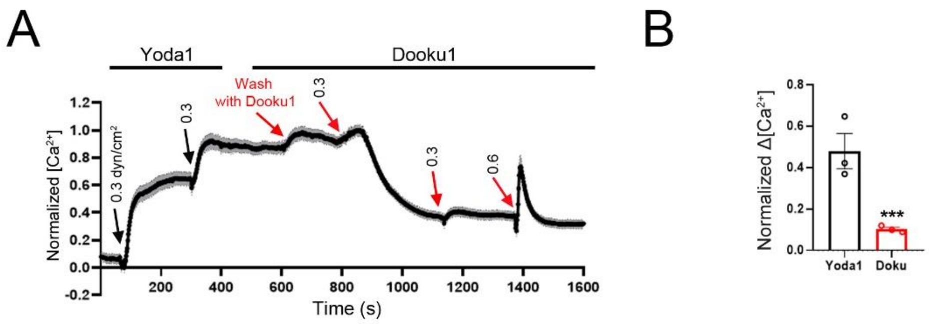

Supplemental Figure 7: The PIEZO1 antagonist, Dooku, blunts the effect of Yoda1 but does not block the response of astrocytes to shear stress, related to Figure 5. (A) Representative trace of normalized change in Ca^{2+} concentration showing the blockade of the Yoda1 shear response after addition of Dooku. Series of 10 sec shear forces was applied in flow medium containing 45 μM BSA and Yoda1 (black arrows), then 10 nM Dooku was added (red arrows) and series of shear forces was applied. Summary of 3 independent

*experiments showing the block of flow-induced stress after Dooku1 incubation upon the addition of Yoda1 at 0.3 dyn/cm^2. Unpaired Mann–Whitney test: ***p < 0. 001. All data shown as mean ± SEM.*

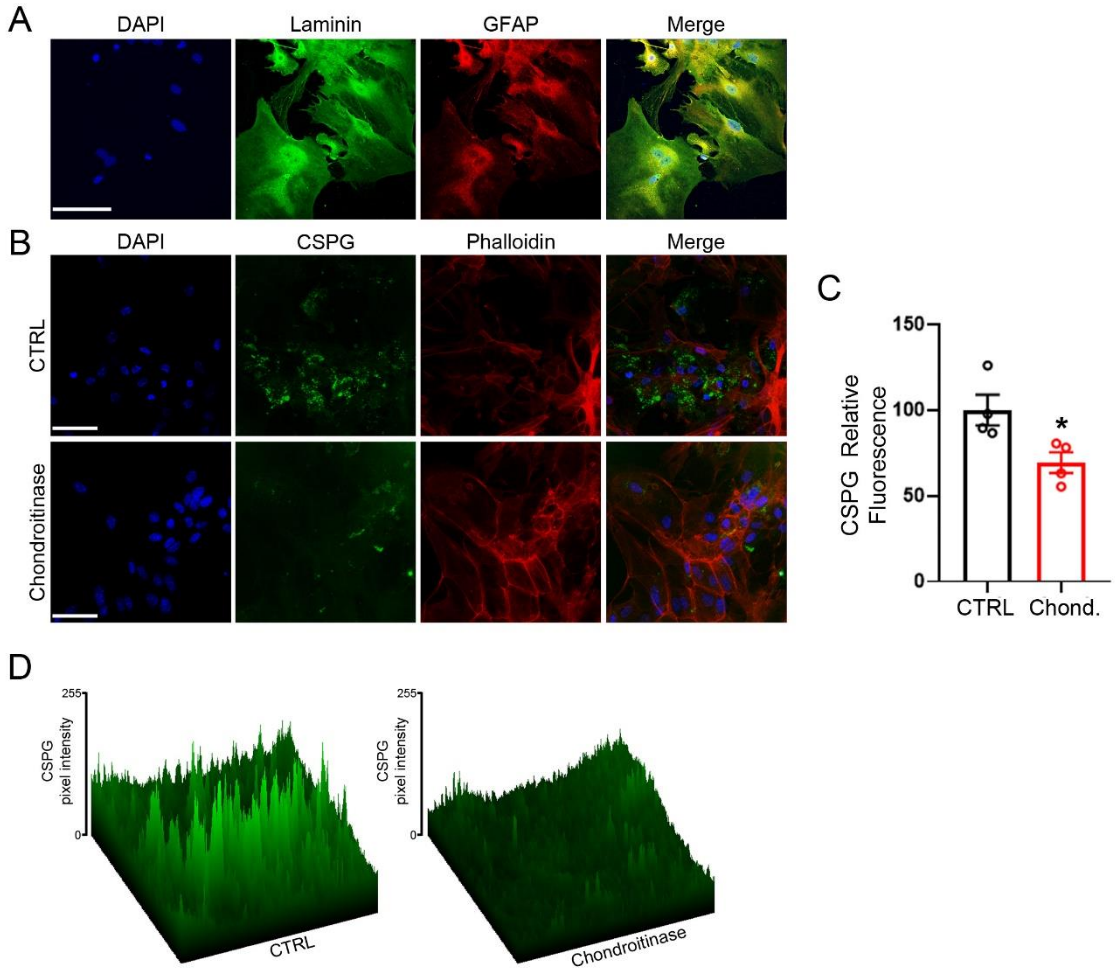

Supplemental Figure 8: Chondroitinase ABC degrades CSPG from the cell surface of mouse astrocytes, related to Figure 7. (A) Representative single-slice confocal images of laminin (green), GFAP (red) and their merge in astrocytes. The nuclei were stained blue with DAPI. Scale bar, 50 µm. (B) Representative single-slice confocal images of CSPG (green), F-actin cytoskeleton visualized with phalloidin (red) and their merge in astrocytes. Cells were cultured in DMEM without (CTRL) and with chondroitinase ABC (Chondroitinase) for 2 h. The nuclei were stained blue with DAPI. Scale bar, 50 µm. (C) Histogram showing

*CSPG fluorescence intensity quantification expressed as a percentage relative to that of the control (CTRL, black), considered as 100% after 2 h treatment with chondroitinase ABC (Chond., red). Data are expressed as mean ± SEM. Four images per experiment, from a total of four experiments, were taken for each condition. Unpaired Student's t test: *$p < 0.05$. (D) Reconstructed 3D surface plot images of (B). Color range from black to green indicates increased level of fluorescence pixel intensity.*
